# Artifact-free ultralow-temperature DNP-enhanced NMR of molecular assemblies at natural isotopic abundance

**DOI:** 10.1126/sciadv.aeb0337

**Published:** 2025-12-19

**Authors:** Quentin Reynard-Feytis, Subhradip Paul, Sabine Hediger, Gaël De Paëpe

**Affiliations:** Université Grenoble Alpes, CEA, CNRS, IRIG, MEM, 38000 Grenoble, France.

## Abstract

Magic-angle spinning dynamic nuclear polarization (MAS-DNP) has greatly increased solid-state NMR sensitivity, enabling multidimensional correlation experiments (e.g., ^13^C–^13^C and ^13^C–^15^N) at natural isotopic abundance. Yet, these experiments often suffer from *t*_1_-noise, an artifact that obscures weak cross-peaks, especially in natural abundance samples. Here, we present a method to suppress *t*_1_-noise in ^13^C–^13^C double-quantum–single-quantum (DQ-SQ) spectra by converting DQ coherences into longitudinal two-spin order (*zz*-terms), enabling selective removal of uncoupled ^13^C magnetization. This *zz*-filter, compatible with both *J*- and dipolar-based sequences, markedly improves the spectral quality on both commercial (100 K) and custom-built helium-spinning (30 K) MAS-DNP setups. Up to a fivefold increase in signal-to-noise ratio in the indirect dimension allows detection of previously hidden long-range correlations, including intermolecular contacts. This method yields the first artifact-free ^13^C–^13^C DQ-SQ spectrum at 30 K, expanding the analytical reach of MAS-DNP NMR for characterizing molecular assemblies at natural abundance.

## Teaser

Artifact-free ultralow-temperature MAS-DNP NMR reveals hidden long-range ^13^C–^13^C correlations at natural abundance.

## INTRODUCTION

Solid-state nuclear magnetic resonance under magic angle spinning (MAS ssNMR) (*1*, *2*) has emerged as a versatile and powerful spectroscopy to study structure and dynamics at the atomic scale of a large variety of systems ranging from biomolecules to materials (*3*–*6*). This was enabled by many methodological and instrumental developments that include access to higher magnetic fields and faster MAS probes and also by the advent of high-power microwave sources (gyrotrons) (*7*, *8*) for the development of high-frequency dynamic nuclear polarization under MAS (MAS-DNP) (*3*, *4*, *7*–*18*). These advancements have markedly broadened the scope of NMR, enabling increasingly challenging applications. Notably, it facilitated two-dimensional (2D) homonuclear and heteronuclear correlation experiments between low-γ/low natural isotopic abundance nuclei (*19*, *20*). As demonstrated in 2012, MAS-DNP applied to systems at natural abundance enables the detection of one bond ^13^C–^13^C correlations (*21*, *22*) and long-range ^13^C–^13^C polarization transfers within hours (*21*, *23*). This strategy has proven key in various contexts, including the study of organic microcrystals (*21*, *22*, *24*), self-assembled peptides and proteins (*20*, *23*, *25*), and the identification of pharmaceutical polymorphs (*26*). In addition, this approach significantly advances the feasibility of biomolecular NMR at natural abundance. Remarkable applications include investigations of amyloid fibrils relevant to Huntington’s disease (*27*); bone-tissue materials, including GAG-collagen interactions in cartilage (*28*, *29*); and ligno-cellulosic materials (*30*–*35*). These developments are particularly important as they overcome longstanding limitations that had severely constrained previous ssNMR studies (*19*). Beyond the primary advantage of eliminating the need of isotopic labeling, which is often costly and not applicable to all systems, working at natural isotopic abundance offers additional inherent benefits. These include simplified spin dynamics and the ability to probe long-range magnetization transfer (*19*–*21*, *23*, *26*, *36*). In 2017, Märker *et al.* (*37*) demonstrated the feasibility of achieving strong agreement between experimental and simulated dipolar build-up curves, arising from the superposition of various distances up to 7 Å in organic solids at natural abundance. In the same year, Kobayashi *et al.* introduced CHHC correlation experiments under MAS-DNP to probe long-range intra- and intermolecular ^13^C–^13^C distances in the same range by exploiting proton-mediated polarization transfer (*36*). The steady increase in MAS-DNP sensitivity over the past decade, driven by advances in polarizing agents (*24*, *38*–*43*) and innovations in hardware that enable experiments at ultralow temperatures with cryogenic helium sample spinning (*12*, *44*–*49*), has significantly enhanced our ability to explore natural-abundance systems with up to two orders of magnitude of sensitivity improvement. However, this increased sensitivity also places greater demands on hardware stability. When experimental reproducibility is insufficient, multidimensional NMR experiments can be affected by a phenomenon known as *t*_1_-noise, which results from fluctuations in the detected signal due to experimental instabilities. In multidimensional NMR experiments, these fluctuations introduce an additional incoherent modulation of the signal along the indirect evolution time (*t*_1_). Because of its multiplicative nature, *t*_1_-noise is proportional to the detected signal intensity (*50*). If *t*_1_-noise exceeds thermal noise, it leads to signal-proportional ridges along the *f*_1_ dimension in the Fourier-transformed spectrum, which can significantly degrade data quality. This issue becomes particularly problematic when cross-peaks of interest are of similar or lower intensity than the *t*_1_-noise, as often encountered in DNP-enhanced correlation experiments involving low abundance nuclei (e.g., ^13^C natural abundance 1.1%). In these cases, *t*_1_-noise can obscure meaningful spectral features, posing a major obstacle to the broader application and further development of multidimensional spectroscopy at natural abundance. Here, we present an original approach to suppress *t*_1_-noise in the context of ^13^C–^13^C double-quantum–single-quantum (DQ-SQ) correlation spectroscopy at natural abundance. It consists in converting DQ coherences (DQCs) into longitudinal two-spin order $I_{\mathrm{jz}}I_{\mathrm{kz}}$ (*zz*-terms) to selectively eliminate, through a “*zz*-filter,” unwanted magnetization of abundant, uncoupled ^13^C nuclei, which constitute the main *t*_1_-noise contribution. This methodology is explained theoretically and demonstrated for both isotropic *J*- (through-bond) and anisotropic dipolar- (through-space) based polarization transfers. Furthermore, we demonstrate that the application of a *zz*-filter together with a simple *z*-filter—referred to as *z*^3^-filter—further reduces the *t*_1_-noise to near the thermal noise level and thereby significantly enhances the overall signal-to-noise ratio (SNR) despite an approximately twofold loss in signal intensity. Using a commercial MAS-DNP setup at 100 K, we first show that spectral clarity is significantly enhanced, enabling straightforward assignment of numerous previously obscured cross-peaks corresponding to long-range correlations (3 to 4 Å), including intermolecular contacts. We then apply the *zz*-filter at 30 K using a custom-built He-spinning MAS-DNP system (*44*), where ultrahigh sensitivity, about one order of magnitude larger than at 100 K due to both larger signal enhancement and reduced thermal noise, leads in more pronounced *t*_1_-noise artifacts. With the approach introduced here, we achieve a remarkable ~5× improvement in SNR along the *f*_1_ dimension, enabling the acquisition of 30 K ^13^C–^13^C DQ-SQ spectrum of small molecules at natural abundance. These results pave the way for more challenging applications at natural abundance with MAS-DNP, including precise measurements of build-up curves for the extraction of long-range interatomic distances in organic and biomolecular solids. Last, we extend the *z*^3^-filter to the heteronuclear context using the transferred echo double resonance (TEDOR) experiment (*51*). Notably, we show that the *zz*-filter, originally implemented by Jaroniec and coworkers to dephase multiple-quantum coherences (MQCs) in ^13^C, ^15^N -labeled system, is equally essential in experiments at natural abundance for the suppression of *t*_1_-noise artifacts.

## RESULTS

### *t*_1_-noise and multidimensional ^13^C–^13^C correlation experiments at natural isotopic abundance

**Fig. 1.**
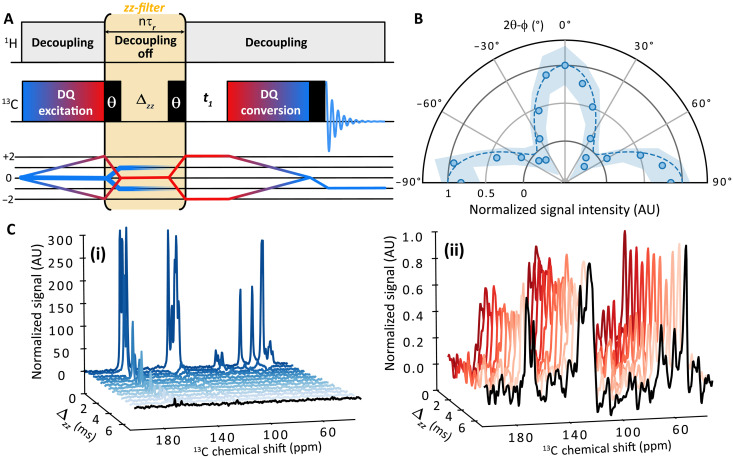
Concept and experimental validation of the zz-filter. (**A**) Schematic of a generic DQ/SQ pulse sequence with the *zz*-filter applied after the DQ-excitation block. The sequence starts with ^13^C *I*_z_ magnetization, which can be prepared by a CP transfer followed by a flip-back 90° pulse (not shown in the sequence). The first ${\left(\pi /2\right)}_{\theta }$ pulse of the *zz*-filter converts half of the DQC into a *zz*-term and uncoupled spin magnetization, *I*_*z*_, into SQC. During $\Delta_{\mathrm{zz}}$, the SQC is dephased, while the *zz*-term is preserved. The second ${\left(\pi /2\right)}_{\theta }$ pulse then converts the *zz*-term into a mix of DQC and ZQC. The coherence transfer pathway (CTP) of the sequence is given below it. The thick blue lines indicate the CTP of uncoupled spins, while the thin blue-red shaded lines are for coupled spin pairs. (**B**) Polar plot of the DQ-filtered normalized signal intensity as a function of the pulse phase θ relative to the phase ϕ of the excited DQC. Blue dots are experimental points acquired with the *J*-INADEQUATE pulse sequences (see figs. S3 and S4A) on ampicillin microcrystal powder, with a *zz*-filter of $25\tau_{r}≃$ 3 ms. Experimental error intervals are represented by the shaded regions. The theoretical efficiency (Eq. 11) is indicated by the dotted blue line. (**C**) Detected 1D signal in a *J*-based DQ/SQ experiment as a function of $\Delta_{\mathrm{zz}}$, with $t_{1}=0$: (i) single-scan signal showing the strong reduction of SQCs with $\Delta_{\mathrm{zz}}$ as a result of signal dephasing and (ii) DQ phase-cycled signal over 64 scans showing conserved DQ-filtered signal intensity during $\Delta_{\mathrm{zz}}$.

**Fig. 2.**
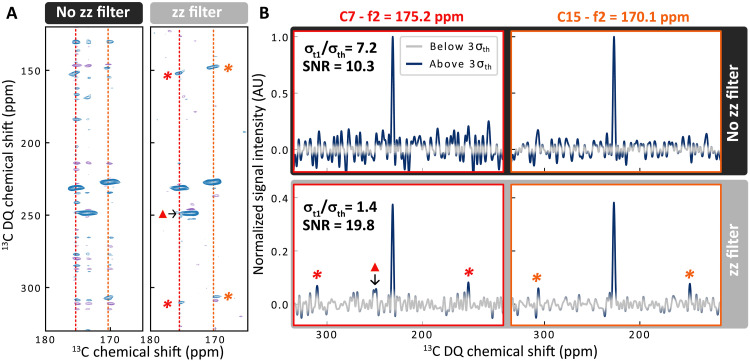
Proof-of-concept demonstration of the *zz*-filter on isotropic *J*-based correlation experiments. (**A**) Carbonyl region of refocused INADEQUATE DNP-enhanced spectra on natural abundance ampicillin microcrystals impregnated with cAsymPol-POK, without (on the left) and with (on the right) a *zz*-filter of 6.25 ms (standard *z*-filter set to 3 ms). Contour levels are normalized to the maximum intensity in each spectrum. (**B**) *f*_1_-traces from (A) at 175.2 and 170.1 ppm [red and orange dotted lines in (A), left and right panels in (B), respectively] without (top panels) and with (bottom panels) *zz*-filter. Intensities are normalized according to the maximum intensity in the corresponding trace without *zz*-filter. The gray region corresponds to noise within three times the SD of the thermal noise $3\sigma_{\text{th}}$. The *t*_1_-noise SD $\sigma_{t1}$ was calculated from regions without any peaks of the *f*_1_-traces (from 0 to 120 ppm and 320 to 400 ppm; see section S3 for more details). Sidebands are indicated by orange and red stars. The orange triangle indicates a real signal corresponding to the foot of a more intense peak. AU, arbitrary unit.

*t*_1_-noise artifacts are typically encountered in multidimensional correlation experiments involving nuclei with low natural isotopic abundance. In these cases, cross-peaks coexist with unwanted signals from uncoupled spins that can be orders of magnitude more intense. Although these stronger signals are typically canceled out using phase cycling, it is well known that minor experimental fluctuations can induce *t*_1_-noise of comparable intensity to the cross-peaks, thereby contaminating the 2D spectrum (*52*). In ^13^C–^13^C correlation experiments at natural abundance, DQ filtration via phase cycling is typically used to remove the large contribution from uncoupled nuclei to detect weaker contributions from coupled spin pairs (Fig. 1, A and C) (*53*, *54*). Even if the latter can largely be removed, its fluctuation from one scan to another can lead to substantial *t*_1_-noise artifacts under DNP conditions. This is especially true when using the latest generation of polarizing agents (*24*, *40*, *43*, *55*) and when experiments are conducted at ultralow temperatures (<100 K) because it increases the signal while decreasing the thermal noise (*44*). Despite excellent experimental stability, the sensitivity can be so high with MAS-DNP that *t*_1_-noise can be present, degrading the spectral quality and preventing unambiguous assignment of weak cross-peaks (see Fig. 2A). While improving instrumental stability is the most direct way to mitigate *t*_1_-noise, identifying the relative contribution of the various potential sources of instabilities (probe detuning, MAS fluctuations, gyrotron instabilities, etc.) and refining the instrumentation accordingly are complex and long-term undertaking, which cannot always be fully achieved. For this reason, additional strategies have been proposed, which generally fall into two main categories: postprocessing methods and improved data-acquisition strategies. Postprocessing methods typically aim to separate the signal from *t*_1_-noise by using the specific characteristics of either the signal or the noise itself, such as the general assumption of *t*_1_-noise homogeneity across the *f*_2_ dimension (i.e., all peaks experience similar fluctuations) (*56*). Consequently, a range of postprocessing methods has been developed since the initial observation of *t*_1_-noise artifacts (*52*, *57*–*65*). While these approaches can be highly beneficial, their applicability can be limited in some contexts by important drawbacks, such as the unintended generation of artificial cross-peaks or the suppression of true ones. On the other hand, experimental approaches tend to provide clearer and more reliable data by minimizing *t*_1_-noise at its source. When *t*_1_-noise results from slow-fluctuating instabilities (such as temperature variations), randomizing the acquisition order of the indirect dimension (*66*) or simply reducing the number of scans for each *t*_1_ increment (*67*) result in its reduction. However, these techniques are ineffective on *t*_1_-noise originating from scan-to-scan instabilities, which disrupt the reproducibility required for effective signal cancellation via phase cycling. This situation is typically encountered in solid-state NMR, where MAS fluctuations are commonly presumed to be the primary source of *t*_1_-noise. Several methods have recently been proposed. Some of these aim at increasing the sequence robustness against MAS fluctuations, e.g., by the design of “γ-free” sequences (*68*) or by refocusing the ^1^H chemical shift anisotropy (CSA) in heteronuclear correlation (HETCOR) experiments (*69*, *70*). Other approaches focus on minimizing the coherences that generate *t*_1_-noise by using selective saturation pulses to suppress specific intense signals (*71*) or removing isolated nuclear spin magnetization in proton-detected HETCOR experiments involving low-isotopic abundance nuclei, either via a *z*-filter (*72*) or more elaborate purging schemes (*69*, *73*). More recently, Perras and coworkers (*74*) proposed a simple approach to limit *t*_1_-noise by shortening the experimental recovery delay. This easy-to-implement solution can in principle be applied to any experiment affected by *t*_1_-noise. However, it comes with a significant loss of sensitivity, particularly when the *t*_1_-noise is much larger than the thermal noise. In addition, the described approach does not account for the recycle-delay limitations of solid-state NMR probes in the presence of high-power decoupling. In liquid-state NMR, coherence selection using pulsed field gradients has proven highly effective for *t*_1_-noise suppression compared to phase-cycled selection (*75*, *76*), although their comparatively lower sensitivity makes them advantageous only when the *t*_1_-noise significantly exceeds the thermal noise level (*76*). While the implementation of gradient coils has been widely adopted in the context of high-resolution magic angle spinning (*77*, *78*), it remains underdeveloped in the context of solid-state NMR (*79*).

### Principles and theory of the zz-filter

Here, we consider the introduction of the *zz*-filter pulse sequence element ${\left(\pi /2\right)}_{\theta }-\Delta_{\mathrm{zz}}-{\left(\pi /2\right)}_{\theta }$ at the end of the DQ excitation block (Fig. 1A), with θ the phase of the two (π/2)-pulses and $\Delta_{\mathrm{zz}}$ the delay between them. Isolated ^13^C nuclear spins, which account for most of the detected signal in 1D cross-polarization (CPMAS) experiments, cannot build any DQCs during this time. Considering the ideal case of radio frequency (rf) field strength perfectly matching the recoupling condition, and the absence of other interactions and rf inhomogeneity, the ^13^C *I_z_* magnetization of isolated spins stays along the *z* axis during the DQ excitation block. The first ${\left(\pi /2\right)}_{\theta }$ pulse of the *zz*-filter brings it in the transverse plane as follows

$$I_{z}\overset{{\left(\pi /2\right)}_{\theta }}{⟶}I_{\theta -\pi /2}$$ (1)

The subsequent delay period $\Delta_{\mathrm{zz}}$ leads to a strong reduction of this isolated spin coherence through its dephasing by chemical shift interactions and heteronuclear couplings to protons (if present). Let us now consider the impact of the *zz*-filter on DQC generated for coupled two-spin system. In the most general case, the DQC created during the DQ excitation block can be defined in the DQ subspace as

$${\text{DQ}}_{\phi }=R_{z}\left(\phi /2\right){\text{DQ}}_{x}R_{z}\left(-\phi /2\right)$$ (2)

where $R_{z}\left(\alpha \right)=e^{-i2{\mathrm{\alpha DQ}}_{z}}$ is the rotation operator about the *z* axis in the DQ subspace, ϕ is the phase of DQC, and the DQ subspace for two coupled spins *j* and *k* is defined by the following operators

$${\text{DQ}}_{x}=\frac{1}{2}\left(2I_{jx}I_{kx}-2I_{jy}I_{ky}\right)=\frac{1}{2}\left(I_{j}^{+}I_{k}^{+}+I_{j}^{-}I_{k}^{-}\right)$$ (3)

$${\text{DQ}}_{y}=\frac{1}{2}\left(2I_{jx}I_{ky}+2I_{jy}I_{kx}\right)=\frac{1}{2i}\left(I_{j}^{+}I_{k}^{+}-I_{j}^{-}I_{k}^{-}\right)$$ (4)

$${\text{DQ}}_{z}=\frac{1}{2}\left(I_{jz}+I_{kz}\right)$$ (5)

If we first consider the case ϕ = 0 for simplicity, the ${\text{DQ}}_{x}$ coherence evolves under the first ${\left(\pi /2\right)}_{\theta }$ pulse of the *zz*-filter as follows (full calculation detailed in the section S8)

$${\text{DQ}}_{x}\overset{{\left(\pi /2\right)}_{\theta }}{⟶}\frac{1}{2}c\left(2\theta \right)\left[{\text{ZQ}}_{x}+{\text{DQ}}_{2\theta }-2I_{jz}I_{kz}\right]-s\left(2\theta \right)\left(I_{j\theta }I_{kz}+I_{jz}I_{k\theta }\right)$$ (6)

with the notation $s\alpha^{n}\equiv \text{sin}{\left(\alpha \right)}^{n}$ and $c\alpha^{n}\equiv \text{cos}{\left(\alpha \right)}^{n}$ and the operators $I_{j\theta }=c\theta I_{\mathrm{jx}}+s\theta I_{\mathrm{jy}}$ and ${\text{ZQ}}_{x}=\frac{1}{2}\left(2I_{\mathrm{jx}}I_{\mathrm{kx}}+2I_{\mathrm{jy}}I_{\mathrm{ky}}\right)$. The subsequent delay $\Delta_{\mathrm{zz}}$ will lead to the dephasing of terms that do not commute with the chemical shift and heteronuclear dipolar interactions. After dephasing, we expect only the *zz*-term to be preserved

$$\overset{\Delta_{\mathrm{zz}}}{\to }-\frac{1}{2}\text{cos}\left(2\theta \right)2I_{jz}I_{kz}$$ (7)

It is now relevant to generalize the result to any DQC with an arbitrary phase ϕ. This can be done using Eq. 2 which, after a variable change θ=θ−ϕ/2, gives the following result

$${\text{DQ}}_{\phi }\overset{{\left(\pi /2\right)}_{\theta }}{⟶}\overset{\Delta_{\mathrm{zz}}}{\to }-\frac{1}{2}\text{cos}\left(2\theta -\phi \right)2I_{jz}I_{kz}$$ (8)

The second ${\left(\pi /2\right)}_{\theta }$ pulse then converts this *zz*-term into a sum of DQ and zero-quantum (ZQ) coherences

$$2I_{jz}I_{kz}\overset{{\left(\pi /2\right)}_{\theta }}{⟶}2I_{j\left(\theta -\pi /2\right)}I_{k\left(\theta -\pi /2\right)}={\text{ZQ}}_{x}-{\text{DQ}}_{2\theta }$$ (9)

Therefore, the effect of the *zz*-filter can be summarized as follows

$${\text{DQ}}_{\phi }\overset{\text{zz}\text{-filter}}{⟶}\frac{1}{2}\text{cos}\left(2\theta -\phi \right)\left({\text{DQ}}_{2\theta }-{\text{ZQ}}_{x}\right)$$ (10)

Projection of ${\text{DQ}}_{2\theta }$ on the initial state before the *zz*-filter (i.e., ${\text{DQ}}_{\phi }$) gives the global efficiency of the *zz*-filter, $\eta_{\mathrm{zz}}$

$$\eta_{\mathrm{zz}}=\frac{1}{2}\text{cos}{\left(2\theta -\phi \right)}^{2}$$ (11)

The *zz*-filter has, therefore, a maximum efficiency of ^1^/_2_, obtained when the phase of the *zz*-filter pulses satisfies the condition

θ≡ϕ/2 mod π/2 (12)

with ϕ the phase of the generated DQC. The corresponding coherence transfer pathway (CTP) is represented on Fig. 1A below the pulse sequence. Overall, in the ideal case where all generated DQCs share the same phase ϕ, the *zz*-filter is expected to substantially suppress signals from uncoupled spins while scaling the intensity of DQ-filtered signal by a factor of 2. This condition is met with *J*-based polarization transfer (e.g., with refocused INADEQUATE), which will serve as a proof of concept to assess the efficiency of the *zz*-filter. Later, we will address the case of dipolar-based transfer, where the anisotropic nature of the dipolar coupling introduces additional complexity. Note that we chose to use the terminology “*zz*-filter” to stress that that the method relies on a two-spin order filtration, although it is clear that the *z*-filter terminology introduced by Sørensen and coworkers in 1984 (*80*) also preserves longitudinal multiple-spin order terms ($2I_{\text{jz}}I_{\text{kz}},4I_{\text{jz}}I_{\text{kz}}I_{\text{lz}}\ldots$). The terminology *zz*-filter has also been used by Pelton and Wemmer for a case specifically aiming at filtering *zz*-terms (*81*). In modern NMR applications, *zz*-filters, which refer to double-isotope filters, have been used in liquid-state NMR on natural abundance molecules, to specifically select ^1^H signal from ^1^H–^13^C spin pairs (*82*–*84*).

### zz-filter in the case of isotropic *J*-based correlation experiments

INADEQUATE is a major NMR experiment that enables probing through-bond homonuclear correlations (*54*, *85*–*87*). It is based on the use of the isotropic *J*-coupling to transfer magnetization between nuclei connected through one or more chemical bonds. Because of the isotropic nature of the *J*-coupling, the phase of the DQC is the same regardless of the crystallite orientation. Following the phases of the pulse sequence used by Cadars and coworkers (see fig. S4A) (*87*), DQCs are generated along ${\text{DQ}}_{y}$ (ϕ=π/2). According to Eq. 11, the maximum efficiency of the *zz*-filter is then expected for pulse phases θ=π/4. This prediction was experimentally verified by measuring the DQ-filtered signal intensity obtained using the *zz*-filtered refocused INADEQUATE sequence, as a function of the *zz*-filter pulse phase θ. The data are plotted as a function of 2θ−ϕ on the polar plot in Fig. 1B (blue dots), together with the analytical expression from Eq. 11 (blue dashed lines), showing excellent agreement within the experimental error bounds (shaded region). The complete pseudo-2D spectrum is provided in the fig. S3. The behavior of uncoupled spins magnetization and of the generated DQCs during the *zz*-filter can be studied by following the signal evolution as a function of the *zz*-filter length and is illustrated on Fig. 1C. Signal from uncoupled spins is strongly dominating when the experiment is acquired with a single-scan (Fig. 1C, i), as CTP selection via phase cycling is then avoided. This strong initial signal, 300-fold more intense than the DQ-filtered signal (with *zz*-filter) usually detected with phase cycling, is dephased during the *zz*-filter and its intensity drastically decreases after a few ms (Fig. 1C, i). On the contrary, the DQ-filtered signal intensity obtained with the complete phase cycling, which aims at removing the strong signal from isolated spins, remains stable throughout the *zz*-filter (Fig. 1C, ii) on the same timescale. It is worth noting that the isolated spin signal of the carbonyls has not fully vanished after 6 ms of *zz*-filter duration, unlike that of the other carbons, as a result of a weaker dipolar coupling to ^1^H for these quaternary carbons. Therefore, we investigated the possibility of actively reintroducing ^1^H–^13^C dipolar interactions during the *zz*-filter using a dipolar-assisted rotational resonance (DARR) recoupling field (*88*) on the ^1^H channel (spin-lock at $\omega_{1H}=n\omega_{r}$ with *n* = 1 or 2). As can be seen in the section S5, it enables a significantly faster decay of the unwanted SQCs but does not improve *t*_1_-noise suppression in this specific case. However, it might be very useful, or even necessary, when using the *zz*-filter under different conditions (sample with a lower ^1^H content, higher MAS frequencies, etc). The efficiency of the *zz*-filter to reduce *t*_1_-noise in DQ/SQ refocused INADEQUATE experiments is illustrated in Fig. 2 using a 6.25-ms *zz*-filter duration. Figure 2A compares spectra acquired without and with *zz*-filter, with contour levels normalized to the maximum intensity in each dataset. The displayed spectra focus on the carbonyl region (see fig. S4B for full spectra). Very clearly, the *zz*-filter leads to a strong reduction of the *t*_1_-noise, resulting in an increased SNR along signal-containing *f*_1_-traces. To evaluate the improvement in SNR, *f*_1_-traces taken at 175.2 parts per million (ppm) (C7) and 170.1 ppm (C15) are compared in Fig. 2B. The gray-colored parts of the spectra correspond to intensities which are below three times the thermal noise standard deviation (SD) $\sigma_{\text{th}}$ (i.e., corresponding to the 99.7% confidence interval of the thermal noise). Further details on this representation are provided in Materials and Methods and section S3. As shown in the top panels of Fig. 2B, for the INADEQUATE experiment without the *zz*-filter, the noise in the extracted *f*_1_-traces significantly exceeds the $3\sigma_{\text{th}}$ threshold due to the presence of *t*_1_-noise. This elevated noise level can obscure genuine peaks and potentially introduce artifacts. In contrast, for the INADEQUATE with *zz*-filter (bottom panels in Fig. 2B), most of the noise falls below the $3\sigma_{\text{th}}$ threshold, indicating that the noise level is now close to the thermal noise limit. This notable reduction in *t*_1_-noise enables the clear identification of several real signals in both the 2D spectra (Fig. 2A) and *f*_1_-traces (Fig. 2B). These signals, marked by stars, were previously hidden in the *t*_1_-noise. The SD of the *t*_1_-noise, $\sigma_{t1}$, was estimated by measuring the SD in a signal-free region of the most contaminated *f*_1_-trace (C7) (see fig. S2A) and was compared to the thermal noise SD $\sigma_{\text{th}}$. The application of the *zz*-filter reduces the $\sigma_{t1}$/$\sigma_{\text{th}}$ ratio from 7.1 to 1.4, bringing the *t*_1_-noise level close to the thermal noise limit. The *zz*-filter efficiency is ~40%, approaching the theoretical maximum of 50%. Consequently, the SNR of the *f*_1_-traces increased from 10.3 to 19.8—an almost twofold enhancement—confirming the effective suppression of *t*_1_-noise. On the other hand, note that the signal loss associated with the *zz*-filter makes this approach advantageous only when the *t*_1_-noise substantially exceeds the thermal noise. This is illustrated in panel (ii) of fig. S2A, where we show an *f*_1_-trace of an aliphatic carbon exhibiting less *t*_1_-noise and a $\sigma_{t1}$/$\sigma_{\text{th}}$ ratio of only 2.3. In this case, although the application of the *zz*-filter further decreases the $\sigma_{t1}$/$\sigma_{\text{th}}$ ratio to 1.1, it also reduces the SNR from 32 to 26.3. It is worth noting that the *J*-refocused INADEQUATE experiment is typically combined with a *z*-filter before detection, as originally proposed by Cadars and coworkers (*87*) to suppress MQCs that may cause lineshape distortions in ^13^C-labeled solids. In natural-abundance samples, however, the strong spin dilution notably limits the formation of these MQCs, and the need for a *z*-filter is, in principle, less critical. Nonetheless, as shown in the section S6), we investigated whether a simple *z*-filter could also contribute to *t*_1_-noise suppression in our experimental conditions. Although some variability in the measured *t*_1_-noise prevented a fully quantitative conclusion, the results suggest that inserting a *z*-filter can indeed help further reduce the *t*_1_-noise level in *J*-refocused INADEQUATE experiments. Consequently, achieving optimal *t*_1_-noise suppression appears to require the combined use of the *zz*- and *z*-filters before detection. In the following section, we show that this combination—referred to as the *z*^3^-filter—is even more crucial in experiments involving long pulse trains, such as those using dipolar recoupling sequences.

### Extension of the zz-filter to the case of dipolar-based correlation experiments

Dipolar-based DQ spectroscopy is particularly interesting to probe spatial proximities and estimate internuclear distances thanks to the distance dependence of the dipolar interaction (*89*). These experiments are typically based on dipolar recoupling sequences that reintroduce the anisotropic dipolar coupling, which is else averaged out by magic angle spinning. In the case of the widely used symmetry-based sequences introduced by Levitt and coworkers (*90*–*92*), the first-order average Hamiltonian (*93*) can be expressed as

$$\begin{array}{c} {\bar{H}}^{\left(1\right)}\left(\Omega_{\text{MR}},t^{0}\right)\\ =|\omega_{\text{MR}}\left(\beta_{\text{MR}}\right)|\text{exp}[-i\phi_{\text{DQ}}\left(\gamma_{\text{MR}},t^{0}\right){\text{DQ}}_{z}\text{]}{\text{DQ}}_{x}\text{exp}[-i\phi_{\text{DQ}}\left(\gamma_{\text{MR}},t^{0}\right){\text{DQ}}_{z}\text{]} \end{array}$$ (13)

with $\Omega_{\text{MR}}=\left(\alpha_{\text{MR}},\beta_{\text{MR}},\gamma_{\text{MR}}\right)$ as the set of Euler angles defining the rotation of the molecular frame into the rotor frame and *t*^0^ as the common time origin for the entire pulse sequence. For these so-called γ-encoded sequences, the phase of the DQ Hamiltonian $\phi_{\text{DQ}}$ is directly proportional to the Euler angle $\gamma_{\text{MR}}$ associated with each crystallite’s orientation (*93*). As a result, the phases of the generated DQCs are uniformly distributed over the DQ subspace. The overall efficiency of the *zz*-filter, denoted $\langle \eta_{\mathrm{zz}}\rangle$, is thus obtained by integrating the filter efficiency $\eta_{\mathrm{zz}}$ (Eq. 11) over all DQCs’ phases ϕ

$$\langle \eta_{\mathrm{zz}}\rangle =\frac{1}{2\pi }\int_{0}^{2\pi }\frac{\text{cos}{\left(2\theta -\phi \right)}^{2}}{2}d\phi =0.25$$ (14)

**Fig. 3.**
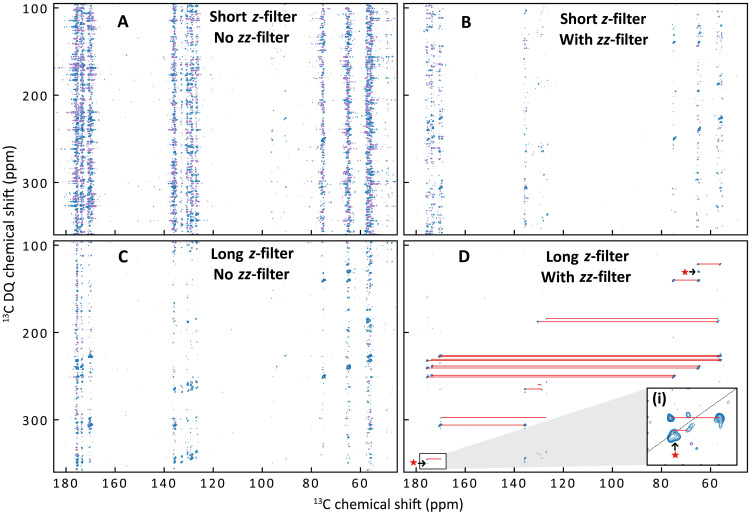
Impact of the *z*- and *zz*-filters on dipolar-based 2D DQ/SQ correlation experiments. 2D DNP-enhanced DQ/SQ SR26 spectra of ampicillin at natural abundance (**A**) without any *zz*-filter and only a short *z*-filter of 100 μs, (**B**) with a *zz*-filter of 5 ms in addition to the short *z*-filter (100 μs), (**C**) with a longer *z*-filter of 3 ms and without *zz*-filter, and (**D**) with both the long *z*-filter (3 ms) and the *zz*-filter of 5 ms. Contour levels are displayed using absolute intensity values and are not normalized to the maximum intensity in each spectrum to accurately reflect the absolute *t*_1_-noise levels. Red lines connect straightforward correlation peaks. Red stars indicate correlations between overlapping or autocorrelation peaks [see zoom (i) on (D)].

**Fig. 4.**
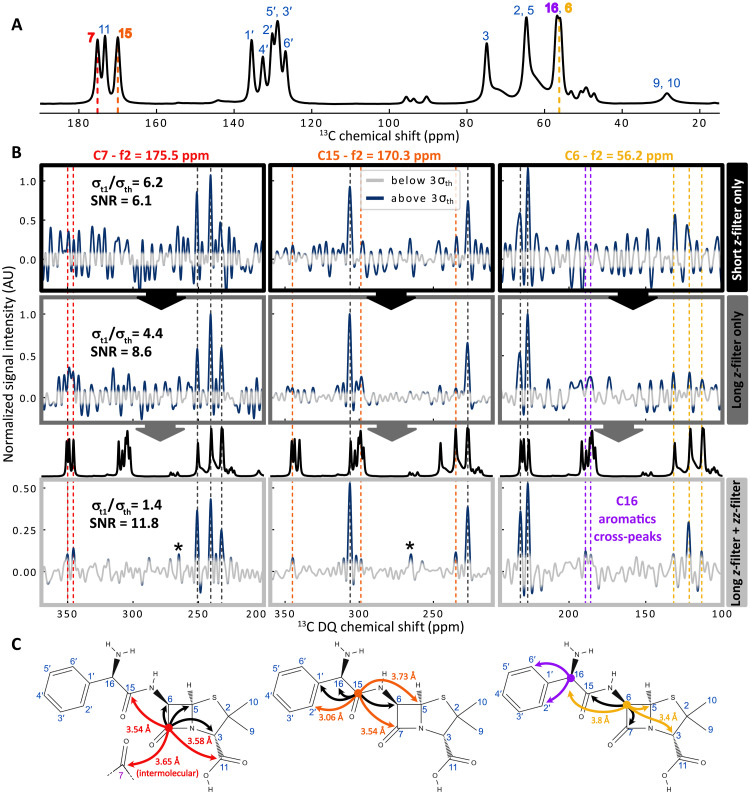
Detailed analysis of the *z*- and *zz*-filters impact in dipolar DQ/SQ correlation experiments. (**A**) 1D ^13^C DNP-enhanced CPMAS spectrum of ampicillin at natural abundance. Assignment is shown in the figure. ^13^C chemical shift positions used in (B) and (C) are highlighted with red (175.2 ppm), orange (170.3 ppm), and yellow (56.2 ppm) dashed lines. (**B**) *f*_1_-traces through dipolar-based DQ/SQ SR26 2D spectra of Fig. 3 at *f*_2_ positions 175.2 ppm (C7, left), 170.3 ppm (C15, middle), and 56.2 ppm (C6, right), without *zz*-filter and with a short *z*-filter of 100 μs (top panels, black frames), with a longer *z*-filter of 3 ms (middle panels, dark gray), and with both 3-ms *z*-filter and 5-ms *zz*-filter (bottom panels, light gray). Each *f*_1_-trace is normalized to the maximum of the *z*-filtered trace to allow intensity comparison. The gray region indicates the thermal noise threshold at $3\sigma_{\text{th}}$. 1D CP spectra (black) shifted according to *f*_2_ positions are overlaid on traces obtained with both filters. Matching cross-peaks are highlighted with dashed lines: dark gray for peaks visible with *z*-filter alone and colored (red, orange, and yellow) for peaks revealed by the *zz*-filter. Sidebands are indicated by black stars. (**C**) Ampicillin chemical structure with arrows illustrating correlations revealed in (B) using the same color code.

Consequently, the *zz*-filter efficiency is reduced to $\langle \eta_{\mathrm{zz}}\rangle =0.25$ for γ-encoded sequences, independently of the *zz*-filter pulses phase θ. On the other hand, the phase of the first-order average Hamiltonian is independent on the γ-angle in non–γ-encoded sequences, such as Baba-xy16 (*94*), $S_{0}S_{0}^{\prime }$ supercycled symmetry-based sequences (*95*) or phase-optimized recoupling with five pi pulses per rotor period (PR5) (*96*). In this case, the *zz*-filter’s efficiency follows the theoretical framework previously introduced and illustrated with the INADEQUATE example, with a theoretical *zz*-filter efficiency of 0.5. This notably applies to supercycled dipolar recoupling sequences such as supercycled R26 (SR26), which are particularly useful for long-distance magnetization transfer (*20*, *95*, *97*, *98*) because of their increased robustness with respect to chemical shift anisotropies and other higher-order average Hamiltonian theory (AHT) contributions (*95*, *97*). The zz-filter efficiency difference for γ- and non–γ-encoded sequences is verified experimentally and computationally [using SIMPSON (*99*, *100*) simulations] in section S7. In Fig. 3, we compare the respective impact of the *z*- (just before direct acquisition) and *zz*-filter (after the SR26 DQ excitation block) in mitigating *t*_1_-noise in DNP-enhanced DQ/SQ SR26 experiments on organic microcrystals at natural abundance. It is worth noting that although SR26 is non–γ-encoded, the sequence can still be implemented with arbitrary spectral width by using the supercycle-timing-compensation (StiC) phase shift, introduced by Märker and coworkers (*98*). Comparing panels (A) with (B) and (C) with D, it is very clear that the *zz*-filter leads to a strong attenuation of the *t*_1_-noise. The *t*_1_-noise is not completely removed with the *zz*-filter alone (see Fig. 3B), highlighting the necessity of combining it with a *z*-filter of sufficient length (3 ms) before detection. This is a noteworthy observation, as it demonstrates that *t*_1_-noise in this experiment arises not only from fluctuations of the longitudinal *I_z_* components of uncoupled nuclei but also from transverse terms. These transverse coherences are generated due to the nonideal behavior of the SR26 excitation block, influenced by the ^13^C CSA, residual proton couplings, and rf inhomogeneities. As a result, transverse coherences can be generated from the *I*_z_ magnetization of isolated ^13^C spins after the excitation block. Even if this signal can be removed by phase cycling and adequate selection of CTP (e.g., DQ filter), its fluctuations can still lead to *t*_1_-noise. While these terms are not suppressed by the *zz*-filter, they can be efficiently attenuated by a sufficiently long *z*-filter that allows their complete dephasing. Therefore, the combined action of the *z*- and *zz*-filters, previously introduced as the *z*^3^-filter, proves highly effective in eliminating the *t*_1_-noise present in this experiment. This is illustrated in Fig. 4B, which compares the *f*_1_-traces extracted from the 2D DQ/SQ SR26 experiments of Fig. 3, taken at 175.5 ppm (C7), 170.3 ppm (C15), and 56.2 ppm (C6), with the corresponding ^13^C assignment shown in Fig. 4A. Noise in *f*_1_-traces from the experiment using only a very short *z*-filter of 100 μs (top black frames of Fig. 4B) is much larger than the thermal noise, whose $3\sigma_{\text{th}}$ interval is highlighted in gray. Only obvious cross-peaks (black dashed lines on Fig. 4B and black arrows in Fig. 4C), corresponding to one- and two-bond correlations (Fig. 4C), can be identified. Any further, less intense cross-peaks originating from longer distance correlation are completely hidden in the *t*_1_-noise, preventing the extraction of any quantitative intensity. Extending the *z*-filter length from 100 μs to 3 ms (middle dark-gray frames) undeniably reduces the *t*_1_-noise, which, however, remains significantly above the thermal noise. Even if additional peaks can be guessed, they are of the same intensity level as the noise, making their interpretation very inaccurate. It is only with the introduction of the *z*^3^-filter (5-ms *zz*-filter and 3-ms *z*-filter) (bottom light-gray frames in Fig. 4B) that the noise of the traces is reduced to the level of the thermal noise, enabling the clear distinction of the small genuine cross-peaks (highlighted with colored dashed lines) from longer distance correlations (colored arrows in Fig. 4C). Most of the unraveled small cross-peaks are assigned to three-bond correlations with corresponding distances of 3 to 4 Å (see Fig. 4C), as expected from the anhydrate ampicillin crystal structure (*101*). One of the peaks on the C7 *f*_1_-trace arises from the overlap between the C7-C11 cross-peak and the C7 autocorrelation peak [see zoom (i) of Fig. 3D]. The latter can be attributed to an intermolecular C7-C7 contact, with a reported distance of 3.65 Å (*101*). As described previously, we quantified the $\sigma_{t1}$/$\sigma_{\text{th}}$ ratio together with the SNR on the *f*_1_-trace of C7, which shows the highest *t*_1_-noise (see fig. S2B for further details). The *zz*-filter efficiency lies in the 45 to 50% range, while the $\sigma_{t1}$/$\sigma_{\text{th}}$ ratio (see Fig. 4B) decreases progressively from 6.2 with a short *z*-filter to 4.4 with a long *z*-filter and down to 1.4 with a *z*^3^-filter. This reduction directly translates into an SNR increase from 6.1 (short *z*-filter) to 8.6 (long *z*-filter) and 11.8 (*z*^3^-filter). Therefore, our experiments demonstrate that combining the *zz*-filter with a sufficiently long *z*-filter—dubbed *z*^3^-filter—significantly reduces *t*_1_-noise and thereby enhances the resulting SNR in dipolar-based DQ/SQ 2D experiments despite the crystallite-orientation dependence of the Hamiltonian.

### *z*^3^-filter DNP-enhanced ^13^C–^13^C natural abundance spectroscopy at ultralow temperature

**Fig. 5.**
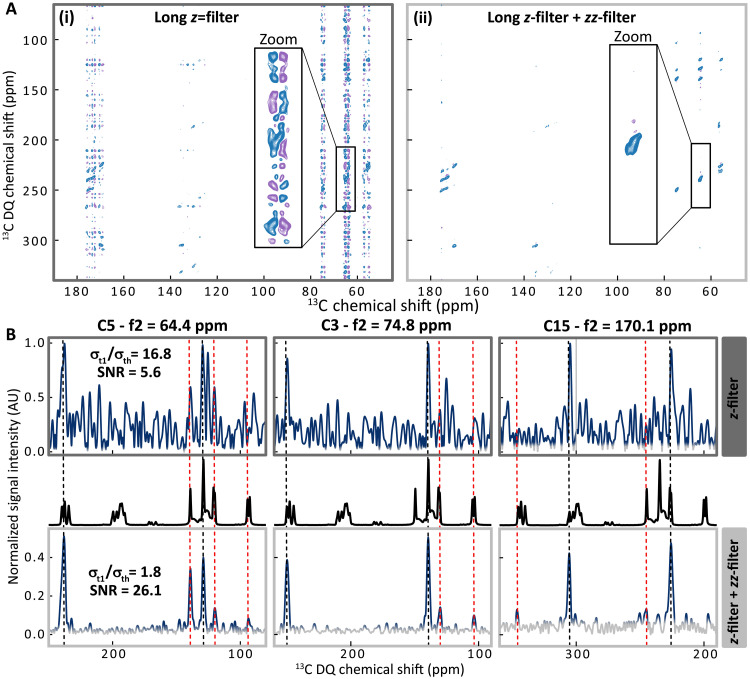
Impact of the *z*^3^-filter on dipolar-based 2D DQ/SQ correlation experiments at 30 K using cryogenic He spinning. (**A**) 2D DNP-enhanced DQ/SQ SR26 spectra of ampicillin at natural abundance with only a *z*-filter of 3 ms (left spectrum with dark-gray frame) and with an additional *zz*-filter of 3 ms as well (i.e., *z*^3^-filter; right spectrum with light-gray frame). Contour levels are normalized to the maximum intensity in each spectrum. (**B**) *f*_1_-traces through the dipolar-based DQ/SQ SR26 2D of (A) at *f*_2_ chemical shift positions of 64.4 ppm (C5), 74.8 ppm (C3), and 170.1 ppm (C15), with the *z*-filter only (top panels with dark gray frames) and with a *z*^3^-filter (bottom panels with light gray frames). To account for the antiphase character of the *t*_1_-noise along the *f*_2_ (direct) dimension under these conditions [see zooms in panels (i) and (ii) of (A)], we chose to display the modulus (absolute value) of the complex spectrum along *f*_2_ while retaining the real part along the indirect dimension (*f*_1_). Each pair of *f*_1_-traces is normalized relative to the maximum intensity in the corresponding *z*-filtered trace (top panels with dark gray frames), such that peak intensities can be compared between both experiments. The gray area in each trace indicates the thermal noise threshold, set at $3\sigma_{\text{th}}$, with $\sigma_{\text{th}}$ estimated assuming a Rayleigh distribution. Further details regarding the representation of the *f*_1_-traces and the determination of $\sigma_{\text{th}}$ are provided in section S3. To help cross-peak identification, 1D CP spectra (in black) shifted according to the corresponding *f*_2_ position are plotted on top of each *f*_1_-trace obtained with the *z*^3^-filter. Cross-peaks revealed by the application of the *zz*-filter are highlighted using red dashed lines.

In the sections above, we have shown that *t*_1_-noise can be present in ^13^C–^13^C natural abundance DNP-enhanced correlation spectra recorded at 100 K using a commercial setup. We expect this issue to get even worse at lower temperature because the thermal noise becomes smaller while the signal can be further enhanced. This hypothesis was tested on an experimental setup developed in house composed of a closed-loop cryogen-free Helium cryostat and an ultralow temperature probe that enables conducting MAS-DNP experiments down to 20 to 30 K. The overall stability of this DNP system with respect to sample spinning and rf/microwave power was discussed previously and is expected to be similar than for the 100 K experiments reported above (*44*). Up to one order of magnitude improvement in sensitivity have been reported previously conducting DNP experiments at 20 to 30 K compared to 100 K (*12*, *44*, *45*, *47*–*49*, *102*). As shown in the 30 K DNP spectrum reported on the left-hand side of Fig. 5A, we observe a significant *t*_1_-noise contribution when using only a *z*-filter of 3 ms. The *t*_1_-noise in the spectrum of Fig. 5A on the left has a clear antiphase character along the *f*_2_ direct detection dimension, significantly different from the shape of the *t*_1_-noise previously observed at 100 K. This observation underlines the variability of *t*_1_-noise depending on the various potential contributions. Impressively, the introduction of a *zz*-filter of 3 ms, in addition to the *z*-filter, markedly reduces the *t*_1_-noise, leading to a spectrum of unprecedented quality obtained under ULT-MAS-DNP conditions (Fig. 5A, right). The comparison of the three *f*_1_-traces in Fig. 5B—taken through both 2D spectra at 64.4 ppm (C5), 74.8 ppm (C3), and 170.1 ppm (C15)—shows that the introduction of the *z*^3^-filter markedly reduces the *t*_1_-noise to the thermal noise level (gray region of the trace). On the *f*_1_-trace of C5, the *zz*-filter efficiency is estimated to be ~50%, while the $\sigma_{t1}/\sigma_{\text{th}}$ ratio decreases from 16.8 to 1.8. This reduction translates into an increase in the SNR from 5.6 to 26.1 when using the *z*^3^-filter, corresponding to an almost fivefold enhancement (Fig. S2C). Further details about the spectrum representation and the measurements of the $\sigma_{t1}/\sigma_{\text{th}}$ and the SNR are provided in Materials and Methods and in section S3).

Similarly to the 100 K case, the elimination of *t*_1_-noise enables the clear and unambiguous assignment of many correlations that were not distinguishable before (red dashed lines). The application of a *z*^3^-filter leads to the first artifact-free DQ/SQ 2D ^13^C–^13^C DNP spectrum recorded at 30 K on a sample at natural abundance.

### Extension of the *z*^3^-filter to the heteronuclear case

Although we focus in this study essentially on homonuclear DQ/SQ correlation experiments, it is interesting to note that the *zz*-filter present in the heteronuclear *z*-filtered TEDOR experiment (*51*) can also be used to mitigate *t*_1_-noise problems. The *zz*-filter was originally introduced together with a *z*-filter before detection to dephase undesired MQCs encountered in spin-labeled spin systems. The authors used a delay between the two 90° pulses that convert $I_{y}S_{z}$- into $I_{z}S_{y}$-terms to preserve *zz*-terms only (see fig. S1). For samples at natural abundance, the formation of these undesired MQCs is very unfavored, and this extra delay is theoretically unnecessary. However, we demonstrate in section S2 that it is still useful to effectively suppress *t*_1_-noise, similarly to the homonuclear case presented above.

## DISCUSSION

In this work, we introduced a method for *t*_1_-noise suppression in MAS-DNP ssNMR experiments in the context of samples at natural abundance. This method, called here *zz*-filter, consists in the conversion of DQCs into longitudinal two-spin order $I_{\text{jz}}I_{\text{kz}}$ (*zz*-terms), while the strong magnetization of uncoupled spins is rotated to the transverse plane. The subsequent *zz*-filter delay leads to the dephasing of the unwanted signal from uncoupled spins by chemical shift and heteronuclear proton interaction terms. The preserved longitudinal two-spin order *zz*-terms are then converted into a mixture of ZQCs and DQCs. The latter follow then the DQ to SQ CTP before being detected. This *zz*-filter is simple to implement (see section S1 for full implementation) and is, in principle, effective regardless of the source of instability leading to *t*_1_-noise. We show that this approach is compatible with both *J*- and dipolar-based DQ/SQ homonuclear correlation experiments, and despite the sole trade-off of an almost twofold loss in signal intensity, it enables a tremendous reduction of the *t*_1_-noise level, leading to spectra of improved sensitivity in the regions of *t*_1_-noise. Demonstrated on a commercial Bruker MAS-DNP system, the combination of the *zz*-filter with a *z*-filter applied before detection, termed *z*^3^-filter, reduces the *t*_1_-noise to the thermal noise level, thereby revealing weak signals from longer-range correlations and even intermolecular contacts that were previously obscured. The performances of the *z*^3^-filter are even more impressive for DNP experiments at ultralow temperature (~30 K), performed on a home-built He-spinning MAS-DNP system, where the increased sensitivity leads to an even more pronounced *t*_1_-noise. The latter is drastically reduced with the application of the *z*^3^-filter, leading to a ~fivefold SNR improvement along the *t*_1_-noise polluted *f*_1_-traces. This approach yields spectra of unprecedented quality under these conditions—at ultralow temperatures and on natural abundance samples—and enables a natural abundance 2D DQ/SQ spectrum recorded at 30 K. This development paves the way for a broad range of applications where *t*_1_-noise has long been the limiting factor, notably the investigation of complex biomolecular and organic systems by ultralow-temperature MAS-DNP and the precise quantification of dipolar build-ups from 2D datasets.

## MATERIALS AND METHODS

### Sample preparation

Ampicillin [d-(-)-α-aminobenzylpenicillin] was purchased from Sigma-Aldrich. Before impregnation, the powder was ground by hand using a mortar to reduce the grain size, and ~45 mg of powder was impregnated with 12 μl of 40 mM cAsymPol-POK (*24*) in d_8_-glycerol/D_2_O/H_2_O (60:30:10, v:v:v). Two samples were prepared from the same stock solution, one packed in a sapphire rotor for the 100 K experiments and another in a zirconia rotor for the 30 K experiments.

### DNP experiments at 100 K

DNP experiments at 100 K were carried out on a Bruker Advance III spectrometer operating at 9.4 T, equipped with a Bruker low temperature 3.2 mm widebore MAS probe and a 263 GHz (9.4 T) gyrotron. With microwave irradiation, the sample temperature was estimated at 106 K. The probe mode was in double resonance mode ^1^H/^13^C for INADEQUATE and SR26 experiments and in triple resonance mode ^1^H/^13^C/^15^N for TEDOR experiments. The spinning speed was set to 8 KHz for most experiments, except for the *zz*-filter efficiency comparison for γ-encoded and non-γ-encoded sequences in Sec. S7, for which the spinning speed was 6.5 kHz.

#### z^3^f-refocused INADEQUATE experiments

the 2D ^13^C–^13^C DQ/SQ *z*-filtered refocused INADEQUATE (*54*, *85*–*87*) spectra presented in Fig. 2 (fig. S4 for the entire spectra) were acquired with 240 increments of 64 scans each using States-time-proportional phase incrementation (TPPI) (*103*) for quadrature detection, a recycle delay of 3 s and 100-kHz swept-frequency two-pulse phase modulation (SWf-TPPM) (*104*–*106*) ^1^H decoupling. Acquisition times were 20 ms for the direct (*f*_2_) and 3 ms for the indirect (*f*_1_) dimension, respectively, leading to an overall experimental time of 13.5 hours. The delay τ was set to 29 rotor periods, corresponding to 3.6 ms (note that the $T_{2}^{\prime }$ on the ampicillin samples was measured at 54.5 ms). As recommended in the zfr-refocused INADEQUATE (*87*), a 3-ms *z*-filter before the detection has been used. The CP contact time was set to 1 ms with a ramped (80 to 100%) CP spin-lock on the ^1^H channel. For the *zz*-filtered experiment, dubbed DQ/SQ *z*^3^-filtered refocused INADEQUATE, the *zz*-filter length was set to 50 rotor periods, corresponding to 6.25 ms, without the application of DARR field on the ^1^H channel. Data were zero-filled before Fourier transform, and a line broadening of 50 Hz was used on the direct dimension. For the results presented in Fig. 1, we used the same sequence with 64 scans [except for Fig. 1C (i) with one scan only], a recycling delay of 4.2 s, and 15 ms of acquisition time. The delay τ was set to 31 rotor periods, which corresponds to 3.9 ms. We also used a *z*-filter of 3 ms, and the *zz*-filter length was set to 25 rotor periods, corresponding to 3.1 ms. Data were zero-filled before Fourier transformed, and a line broadening of 150 Hz was used on the 1D spectra of Fig. 1C (i) for better visualization. For Fig. 1B, the phase ϕ of the generated DQCs was fixed to π/4 according to the phases used in (*87*), and we varied the phase of the *zz*-filter pulses, θ. The resulting pseudo-2D spectrum is represented in fig. S3, and the normalized signal intensity represented in Fig. 1B (blue dots) was obtained by taking the integral of the DQ-filtered signal for each angle increment on the region represented by red-shaded regions. The signal intensities were then normalized by the intensity obtained for 2θ−ϕ=0.

#### z^3^f-SR26 experiments

the 2D ^13^C–^13^C DQ/SQ dipolar correlation experiments of Figs. 3 and 4 were performed with the SR26 recoupling sequence (*95*, *97*) combined with StiC phase shifts (*98*). They were acquired with 240 increments of 16 scans each using the States-TPPI for the quadrature detection, and a recycle delay of 4.5 s, leading to an overall experimental time of 4.5 hours. For both the DQ excitation and reconversion blocks, one cycle of SR26 was used, corresponding to an overall mixing time of 4 ms, with 100-kHz continuous wave–Lee Goldburg (CW-LG) (*107*) ^1^H decoupling. A 100-kHz SWf-TPPM ^1^H decoupling was applied during both the direct and indirect evolution time of 15 and 3 ms, respectively. The CP contact time was set to 2 ms with a ramped (80 to 100%) CP spin-lock on the ^1^H channel. For the *zz*-filtered experiment, the *zz*-filter length was set 40 rotor periods, corresponding to 5 ms, under a DARR field of ~8 kHz on the ^1^H channel ($\omega_{1H}=\omega_{r}$). The phases of the *zz*-filter were incremented for the States-TPPI quadrature detection along with the SR26 reconversion block to filter the DQCs. The data were zero-filled before Fourier transform, without any apodization.

#### z^3^f-TEDOR experiments

the 2D zfr-TEDOR (*51*) spectra presented in fig. S1 were acquired with 64 increments of 128 scans each using Stats-TPPI for quadrature detection, a recycle delay of 4 s and 100-kHz SWf-TPPM ^1^H decoupling. Acquisition times were 8 ms for the direct (*f*_2_) and 4 ms for the indirect (*f*_1_) dimension, respectively, leading to an overall experimental time of 9.5 hours. The mixing time was set to 4 ms, which corresponds to four TEDOR block, during which a 100-kHz CW-LG decoupling was applied on the ^1^H channel. For both experiments, we used a *z*-filter of 3 ms before detection, and we added—for the *zz*-filtered TEDOR—a *zz*-filter of 24 rotor period, corresponding to ~3 ms. We did not use any DARR field on the ^1^H channel during the *zz*-filter. The CP contact time was set to 2 ms with a ramped (80 to 100%) CP spin-lock on the ^1^H channel. The data were zero-filled before Fourier transform, without any apodization.

### DNP experiments at 30 K

DNP experiments at 30 K were carried out on a Bruker Advance III spectrometer operating at 9.4 T, equipped with a 263 GHz (9.4 T) gyrotron and a self-developed closed-loop cryogenic He-spinning system, made of a cryogenic ultralow-temperature 3.2-mm wide bore MAS probe in double resonance mode ^1^H/^13^C and its associated cryostat (*44*). With microwave irradiation, the sample temperature was estimated at 30 K. The spinning speed was set to 6850 Hz. 2D ^13^C–^13^C DQ/SQ dipolar correlation experiments of Fig. 5 were performed with the SR26 recoupling sequence combined with StiC phase shifts. They were acquired with 256 increments of 16 scans, each using the States-TPPI for the quadrature detection, and a recycle delay of 4.5 s, leading to an overall experimental time of 5 hours. For both the DQ excitation and reconversion blocks, one cycle of SR26 was used, corresponding to an overall mixing time of 4.7 ms, with 100-kHz CW-LG ^1^H decoupling. A 100-kHz SWf-TPPM ^1^H decoupling was applied during both the direct and indirect evolution time of 20 and 3.2 ms, respectively. The CP contact time was set to 2 ms with a ramped (80 to 100%) CP spin-lock on the ^1^H channel. For the *zz*-filtered experiment, the *zz*-filter length was set 30 rotor periods, corresponding to 4.3 ms, without any DARR field on the ^1^H channel. The phases of the *zz*-filter were incremented for the States-TPPI quadrature detection along with the SR26 reconversion block to filter the DQCs. The data were zero-filled before Fourier transform, without any apodization.

### Noise analysis

**Fig. 6.**
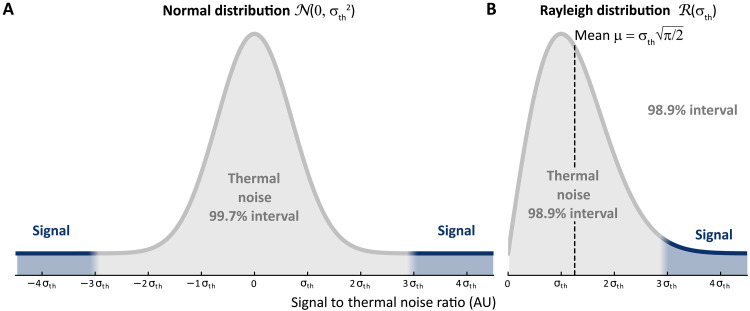
Color gradient representation of *f*_1_-traces based on $\sigma_{\text{th}}$. Schema of the color gradient used to represent *f*_1_-traces based on the thermal noise standard deviation $\sigma_{\text{th}}$ in the case of a normal distribution $N\left(0,\sigma_{\text{th}}\right)$ (**A**) and a Rayleigh distribution $R\left(\sigma_{\text{th}}\right)$ (**B**).

The noise analysis was performed assuming a normal distribution N(0,σ) centered at zero, where σ denotes the noise SD. The thermal noise SD, $\sigma_{\text{th}}$, was measured directly from signal-free *f*_1_-traces at several positions in the spectra (*f*_2_ = −100, −50, 0, 225, 250, 275, and 300 ppm, for all experiments). When displaying *f*_1_-traces from various spectra (Figs. 2 and 4), we used a gray to dark blue color gradient to represent the 99.7% confidence interval of thermal noise, corresponding to the $\pm 3\sigma_{\text{th}}$ limit. The color scale used in Figs. 2 and 4 is illustrated in Fig. 6A. For the 30 K MAS-DNP measurements, a zoom in Fig. 5 shows that the *t*_1_-noise in the 2D spectrum is predominantly antiphase along the *f*_2_ dimension. Because the *f*_1_-trace representation does not reflect this behavior, we instead displayed the data as the absolute value along *f*_2_ and the real part along *f*_1_. Assuming that the real and imaginary components of the signal are independent and follow $N\left(0,\sigma_{\text{th}}\right)$, the corresponding magnitude spectrum follows a Rayleigh distribution $ℛ\left(\sigma_{\text{th}}\right)$. In this case, the noise SD $\sigma_{\text{th}}$ can be estimated from the measured mean noise amplitude μ using

$$\sigma_{\text{th}}=\mu \sqrt{2/\pi }$$ (15)

To quantitatively assess the *t*_1_-noise reduction achieved by the *z*^3^-filter, we estimated the *t*_1_-noise standard deviation $\sigma_{t1}$ from the most noise-polluted *f*_1_-trace, using signal-free regions (highlighted in red in Figure S2). The ratio $\sigma_{t1}/\sigma_{\text{th}}$ thus reflects the relative amplitude of *t*_1_-noise with respect to thermal noise, together with the SNR along the *f*_1_-traces and the *zz*-filter efficiency $\eta_{\mathrm{zz}}$. These values, along with the regions used for their measurement, are shown in fig. S2.

### Practical aspects of the *zz*-filter implementation

In all experiments presented here, the *zz*-filter was applied immediately after the DQ-excitation block and consisted of two 90° pulses with the same phase, separated by a delay $\Delta_{\mathrm{zz}}$. The phases of these pulses were incremented together with the DQ-excitation block to achieve DQ selection via phase cycling or to enable States-TPPI quadrature detection. If phase cycling and/or States-TPPI quadrature detection is encoded on the reconversion block, the *zz*-filter pulse phases should not be incremented. The delay $\Delta_{\mathrm{zz}}$ was generally set to maintain rotor synchronization. While rotor synchronicity is not strictly required in every case, it simplifies implementation without affecting performance. Detailed discussions on *zz*-filter implementation, rotor synchronicity, and optimization can be found in section S1.

## Supplementary Materials

### This PDF file includes:

Sections S1 to S8 Figs. S1 to S7 References

## References

[1] E. R. Andrew, A. Bradbury, R. G. Eades, Nuclear magnetic resonance spectra from a crystal rotated at high speed. Nature 182, 1659–1659 (1958).

[2] I. J. Lowe, Free induction decays of rotating solids. Phys. Rev. Lett. 2, 285–287 (1959).

[3] K. Jaudzems, T. Polenova, G. Pintacuda, H. Oschkinat, A. Lesage, DNP NMR of biomolecular assemblies. J. Struct. Biol. 206, 90–98 (2019). 30273657 10.1016/j.jsb.2018.09.011

[4] A. G. M. Rankin, J. Trébosc, F. Pourpoint, J.-P. Amoureux, O. Lafon, Recent developments in MAS DNP-NMR of materials. Solid State Nucl. Magn. Reson. 101, 116–143 (2019). 31189121 10.1016/j.ssnmr.2019.05.009

[5] B. Reif, S. E. Ashbrook, L. Emsley, M. Hong, Solid-state NMR spectroscopy. Nat. Rev. Methods Primers 1, 2 (2021). 34368784 10.1038/s43586-020-00002-1 PMC8341432

[6] C. P. Gordon, L. Lätsch, C. Copéret, Nuclear magnetic resonance: A spectroscopic probe to understand the electronic structure and reactivity of molecules and materials. J. Phys. Chem. Lett. 12, 2072–2085 (2021). 33617260 10.1021/acs.jpclett.0c03520

[7] L. R. Becerra, G. J. Gerfen, R. J. Temkin, D. J. Singel, R. G. Griffin, Dynamic nuclear polarization with a cyclotron resonance maser at 5 T. Phys. Rev. Lett. 71, 3561–3564 (1993). 10055008 10.1103/PhysRevLett.71.3561

[8] D. A. Hall, D. C. Maus, G. J. Gerfen, S. J. Inati, L. R. Becerra, F. W. Dahlquist, R. G. Griffin, Polarization-enhanced NMR spectroscopy of biomolecules in frozen solution. Science 276, 930–932 (1997). 9139651 10.1126/science.276.5314.930

[9] A. B. Barnes, G. De Paëpe, P. C. A. van der Wel, K.-N. Hu, C.-G. Joo, V. S. Bajaj, M. L. Mak-Jurkauskas, J. R. Sirigiri, J. Herzfeld, R. J. Temkin, R. G. Griffin, High-field dynamic nuclear polarization for solid and solution biological NMR. Appl. Magn. Reson. 34, 237–263 (2008). 19194532 10.1007/s00723-008-0129-1 PMC2634864

[10] A. J. Rossini, A. Zagdoun, M. Lelli, A. Lesage, C. Copéret, L. Emsley, Dynamic nuclear polarization surface enhanced NMR spectroscopy. Acc. Chem. Res. 46, 1942–1951 (2013). 23517009 10.1021/ar300322x

[11] D. Lee, S. Hediger, G. De Paëpe, Is solid-state NMR enhanced by dynamic nuclear polarization? Solid State Nucl. Magn. Reson. 66-67, 6–20 (2015). 25779337 10.1016/j.ssnmr.2015.01.003

[12] D. Lee, E. Bouleau, P. Saint-Bonnet, S. Hediger, G. De Paëpe, Ultra-low temperature MAS-DNP. J. Magn. Reson. 264, 116–124 (2016). 26920837 10.1016/j.jmr.2015.12.010

[13] D. Lee, F. Mentink-Vigier, “Dynamic nuclear polarization for solid-state NMR spectroscopy,” in *Modern NMR Crystallography: Concepts and Applications* (Royal Society of Chemistry, 2025), vol. 36, 256–308.

[14] B. Corzilius, High-field dynamic nuclear polarization. Annu. Rev. Phys. Chem. 71, 143–170 (2020). 32074473 10.1146/annurev-physchem-071119-040222

[15] R. W. Hooper, B. A. Klein, V. K. Michaelis, Dynamic nuclear polarization (DNP) 101: A new era for materials. Chem. Mater. 32, 4425–4430 (2020).

[16] S. Hediger, D. Lee, F. Mentink-Vigier, G. de Paëpe, MAS-DNP enhancements: Hyperpolarization, depolarization, absolute sensitivity. eMagRes 7, 105–116 (2018).

[17] Q. Z. Ni, E. Daviso, T. V. Can, E. Markhasin, S. K. Jawla, T. M. Swager, R. J. Temkin, J. Herzfeld, R. G. Griffin, High frequency dynamic nuclear polarization. Acc. Chem. Res. 46, 1933–1941 (2013). 23597038 10.1021/ar300348n PMC3778063

[18] R. G. Griffin, T. M. Swager, R. J. Temkin, High frequency dynamic nuclear polarization: New directions for the 21st century. J. Magn. Reson. 306, 128–133 (2019). 31327537 10.1016/j.jmr.2019.07.019 PMC6703937

[19] A. N. Smith, K. Märker, S. Hediger, G. De Paëpe, Natural isotopic abundance ^13^C and ^15^N multidimensional solid-state NMR enabled by dynamic nuclear polarization. J. Phys. Chem. Lett. 10, 4652–4662 (2019). 31361489 10.1021/acs.jpclett.8b03874

[20] K. Märker, M. Pingret, J.-M. Mouesca, D. Gasparutto, S. Hediger, G. De Paëpe, A new tool for NMR crystallography: Complete ^13^C/^15^N assignment of organic molecules at natural isotopic abundance using DNP-enhanced solid-state NMR. J. Am. Chem. Soc. 137, 13796–13799 (2015). 26485326 10.1021/jacs.5b09964

[21] H. Takahashi, D. Lee, L. Dubois, M. Bardet, S. Hediger, G. De Paëpe, Rapid natural-abundance 2D ^13^C-^13^C correlation spectroscopy using dynamic nuclear polarization enhanced solid-state NMR and matrix-free sample preparation. Angew. Chem. Int. Ed. Engl. 51, 11766–11769 (2012). 23081784 10.1002/anie.201206102

[22] A. J. Rossini, A. Zagdoun, F. Hegner, M. Schwarzwälder, D. Gajan, C. Copéret, A. Lesage, L. Emsley, Dynamic nuclear polarization NMR spectroscopy of microcrystalline solids. J. Am. Chem. Soc. 134, 16899–16908 (2012). 22967206 10.1021/ja308135r

[23] H. Takahashi, B. Viverge, D. Lee, P. Rannou, G. De Paëpe, Towards structure determination of self-assembled peptides using dynamic nuclear polarization enhanced solid-state NMR spectroscopy. Angew. Chem. Int. Ed. Engl. 52, 6979–6982 (2013). 23564735 10.1002/anie.201210093

[24] R. Harrabi, T. Halbritter, F. Aussenac, O. Dakhlaoui, J. van Tol, K. K. Damodaran, D. Lee, S. Paul, S. Hediger, F. Mentink-Vigier, S. T. Sigurdsson, G. De Paëpe, Highly efficient polarizing agents for MAS-DNP of proton-dense molecular solids. Angew. Chem. Int. Ed. Engl. 61, e202114103 (2022). 35019217 10.1002/anie.202114103 PMC8901535

[25] H. Takahashi, S. Hediger, G. De Paëpe, Matrix-free dynamic nuclear polarization enables solid-state NMR ^13^C-^13^C correlation spectroscopy of proteins at natural isotopic abundance. Chem. Commun. 49, 9479–9481 (2013). 10.1039/c3cc45195j 24013616

[26] G. Mollica, M. Dekhil, F. Ziarelli, P. Thureau, S. Viel, Quantitative structural constraints for organic powders at natural isotopic abundance using dynamic nuclear polarization solid-state NMR spectroscopy. Angew. Chem. Int. Ed. Engl. 54, 6028–6031 (2015). 25809550 10.1002/anie.201501172

[27] A. N. Smith, K. Märker, T. Piretra, J. C. Boatz, I. Matlahov, R. Kodali, S. Hediger, P. C. A. van der Wel, G. De Paëpe, Structural fingerprinting of protein aggregates by dynamic nuclear polarization-enhanced solid-state NMR at natural isotopic abundance. J. Am. Chem. Soc. 140, 14576–14580 (2018). 30339373 10.1021/jacs.8b09002 PMC6287890

[28] S. Wi, N. Dwivedi, R. Dubey, F. Mentink-Vigier, N. Sinha, Dynamic nuclear polarization-enhanced, double-quantum filtered ^13^C-^13^C dipolar correlation spectroscopy of natural ^13^C abundant bone-tissue biomaterial. J. Magn. Reson. 335, 107144 (2022). 35085899 10.1016/j.jmr.2022.107144 PMC8823282

[29] N. Dwivedi, B. Patra, F. Mentink-Vigier, S. Wi, N. Sinha, Unveiling charge-pair salt-bridge interaction between GAGs and collagen protein in cartilage: Atomic evidence from DNP-enhanced ssNMR at natural isotopic abundance. J. Am. Chem. Soc. 146, 23663–23668 (2024). 38980938 10.1021/jacs.4c05539 PMC11572119

[30] F. A. Perras, H. Luo, X. Zhang, N. S. Mosier, M. Pruski, M. M. Abu-Omar, Atomic-level structure characterization of biomass pre- and post-lignin treatment by dynamic nuclear polarization-enhanced solid-state NMR. J. Phys. Chem. A 121, 3, 623–630 (2017). 28026949 10.1021/acs.jpca.6b11121

[31] A. Kirui, Z. Ling, X. Kang, M. C. Dickwella Widanage, F. Mentink-Vigier, A. D. French, T. Wang, Atomic resolution of cotton cellulose structure enabled by dynamic nuclear polarization solid-state NMR. Cellul. 26, 329–339 (2019). 10.1007/s10570-018-2095-6 PMC6615758 31289425

[32] A. Kumar, B. Watbled, I. Baussanne, S. Hediger, M. Demeunynck, G. De Paëpe, Optimizing chemistry at the surface of prodrug-loaded cellulose nanofibrils with MAS-DNP. Commun. Chem. 6, 58 (2023). 36977767 10.1038/s42004-023-00852-2 PMC10049993

[33] A. Kumar, H. Durand, E. Zeno, C. Balsollier, B. Watbled, C. Sillard, S. Fort, I. Baussanne, N. Belgacem, D. Lee, S. Hediger, M. Demeunynck, J. Bras, G. De Paëpe, The surface chemistry of a nanocellulose drug carrier unravelled by MAS-DNP. Chem. Sci. 11, 3868–3877 (2020). 34122855 10.1039/c9sc06312a PMC8152408

[34] P. Berruyer, M. Gericke, P. Moutzouri, D. Jakobi, M. Bardet, L. Karlson, S. Schantz, T. Heinze, L. Emsley, Advanced characterization of regioselectively substituted methylcellulose model compounds by DNP enhanced solid-state NMR spectroscopy. Carbohydr. Polym. 262, 117944 (2021). 33838821 10.1016/j.carbpol.2021.117944

[35] F. Deligey, M. A. Frank, S. H. Cho, A. Kirui, F. Mentink-Vigier, M. T. Swulius, B. T. Nixon, T. Wang, Structure of in vitro-synthesized cellulose fibrils viewed by cryo-electron tomography and ^13^C natural-abundance dynamic nuclear polarization solid-state NMR. Biomacromolecules 23, 2290–2301 (2022). 35341242 10.1021/acs.biomac.1c01674 PMC9198983

[36] T. Kobayashi, I. I. Slowing, M. Pruski, Measuring long-range ^13^C-^13^C correlations on a surface under natural abundance using dynamic nuclear polarization-enhanced solid-state nuclear magnetic resonance. J. Phys. Chem. C 121, 24687–24691 (2017).

[37] K. Märker, S. Paul, C. Fernández-de-Alba, D. Lee, J.-M. Mouesca, S. Hediger, G. De Paëpe, Welcoming natural isotopic abundance in solid-state NMR: Probing π-stacking and supramolecular structure of organic nanoassemblies using DNP. Chem. Sci. 8, 974–987 (2017). 28451235 10.1039/c6sc02709a PMC5354064

[38] C. Sauvée, M. Rosay, G. Casano, F. Aussenac, R. T. Weber, O. Ouari, P. Tordo, Highly efficient, water-soluble polarizing agents for dynamic nuclear polarization at high frequency. Angew. Chem. Int. Ed. Engl. 52, 10858–10861 (2013). 23956072 10.1002/anie.201304657

[39] F. Mentink-Vigier, S. Paul, D. Lee, A. Feintuch, S. Hediger, S. Vega, G. De Paëpe, Nuclear depolarization and absolute sensitivity in magic-angle spinning cross effect dynamic nuclear polarization. Phys. Chem. Chem. Phys. 17, 21824–21836 (2015). 26235749 10.1039/c5cp03457d

[40] F. Mentink-Vigier, I. Marin-Montesinos, A. P. Jagtap, T. Halbritter, J. van Tol, S. Hediger, D. Lee, S. T. Sigurdsson, G. De Paëpe, Computationally assisted design of polarizing agents for dynamic nuclear polarization enhanced NMR: The AsymPol family. J. Am. Chem. Soc. 140, 11013–11019 (2018). 30095255 10.1021/jacs.8b04911 PMC6145599

[41] A. Lund, G. Casano, G. Menzildjian, M. Kaushik, G. Stevanato, M. Yulikov, R. Jabbour, D. Wisser, M. Renom-Carrasco, C. Thieuleux, F. Bernada, H. Karoui, D. Siri, M. Rosay, I. V. Sergeyev, D. Gajan, M. Lelli, L. Emsley, O. Ouari, A. Lesage, TinyPols: A family of water-soluble binitroxides tailored for dynamic nuclear polarization enhanced NMR spectroscopy at 18.8 and 21.1 T. Chem. Sci. 11, 2810–2818 (2020). 34084341 10.1039/c9sc05384k PMC8157490

[42] R. Yao, D. Beriashvili, W. Zhang, S. Li, A. Safeer, A. Gurinov, A. Rockenbauer, Y. Yang, Y. Song, M. Baldus, Y. Liu, Highly bioresistant, hydrophilic and rigidly linked trityl-nitroxide biradicals for cellular high-field dynamic nuclear polarization. Chem. Sci. 13, 14157–14164 (2022). 36540821 10.1039/d2sc04668g PMC9728575

[43] R. Wei, G. Casano, Y. Zhang, I. M. Gierbolini-Colón, Y. Rao, S. S. Gunaga, F. J. Scott, J. Zhou, S. Chatterjee, S. Kumari, H. Karoui, M. Huang, S. T. Sigurdsson, G. De Paëpe, Y. Liu, F. Mentink-Vigier, A. Venkatesh, O. Ouari, L. Emsley, Systematic evaluation of polarizing agents for dynamic nuclear polarization enhanced NMR. Angew. Chem. Int. Ed. 64, e202505944 (2025). 10.1002/anie.202505944 PMC13244539 40418626

[44] S. Paul, E. Bouleau, Q. Reynard-Feytis, J.-P. Arnaud, F. Bancel, B. Rollet, P. Dalban-Moreynas, C. Reiter, A. Purea, F. Engelke, S. Hediger, G. De Paëpe, Sustainable and cost-effective MAS DNP-NMR at 30 K with cryogenic sample exchange. J. Magn. Reson. 356, 107561 (2023). 37837749 10.1016/j.jmr.2023.107561

[45] Y. Matsuki, S. Nakamura, S. Fukui, H. Suematsu, T. Fujiwara, Closed-cycle cold helium magic-angle spinning for sensitivity-enhanced multi-dimensional solid-state NMR. J. Magn. Reson. 259, 76–81 (2015). 26302269 10.1016/j.jmr.2015.08.003

[46] F. Hobo, Y. Tanimoto, Y. Endo, Y. Matsuki, H. Takahashi, 400 MHz/263 GHz ultra-low temperature MAS-DNP using a closed-cycle helium gas cooling system and a solid-state microwave source. J. Magn. Reson. 373, 107842 (2025). 39946944 10.1016/j.jmr.2025.107842

[47] Y. Matsuki, S. Nakamura, F. Hobo, Y. Endo, H. Takahashi, H. Suematsu, T. Fujiwara, Cryogenic signal amplification combined with helium-temperature MAS DNP toward ultimate NMR sensitivity at high field conditions. J. Magn. Reson. 335, 107139 (2022). 34974207 10.1016/j.jmr.2021.107139

[48] Y. Matsuki, T. Fujiwara, “Cryogenic platforms and optimized DNP sensitivity,” in *eMagRes* (John Wiley & Sons Ltd, 2018), pp. 9–24.

[49] K. Thurber, R. Tycko, Low-temperature dynamic nuclear polarization with helium-cooled samples and nitrogen-driven magic-angle spinning. J. Magn. Reson. 264, 99–106 (2016). 26920835 10.1016/j.jmr.2016.01.011 PMC4769783

[50] A. F. Mehlkopf, D. Korbee, T. A. Tiggelman, R. Freeman, Sources of *t*_1_ noise in two-dimensional NMR. J. Magn. Reson. 58, 315–323 (1984).

[51] C. P. Jaroniec, C. Filip, R. G. Griffin, 3D TEDOR NMR Experiments for the simultaneous measurement of multiple carbon-nitrogen distances in uniformly ^13^C,^15^N-labeled solids. J. Am. Chem. Soc. 124, 10728–10742 (2002). 12207528 10.1021/ja026385y

[52] G. A. Morris, H. Barjat, T. J. Horne, Reference deconvolution methods. Prog. Nucl. Magn. Reson. Spectrosc. 31, 197–257 (1997).

[53] A. Wokaun, R. R. Ernst, Selective detection of multiple quantum transitions in NMR by two-dimensional spectroscopy. Chem. Phys. Lett. 52, 407–412 (1977).

[54] A. Bax, R. Freeman, T. A. Frenkiel, An NMR technique for tracing out the carbon skeleton of an organic molecule. J. Am. Chem. Soc. 103, 2102–2104 (1981).

[55] T. Halbritter, R. Harrabi, S. Paul, J. van Tol, D. Lee, S. Hediger, S. T. Sigurdsson, F. Mentink-Vigier, G. De Paëpe, PyrroTriPol: A semi-rigid trityl-nitroxide for high field dynamic nuclear polarization. Chem. Sci. 14, 3852–3864 (2023). 37035686 10.1039/d2sc05880d PMC10074417

[56] G. Bodenhausen, P. H. Bolton, Elimination of flip-angle effects in two-dimensional NMR spectroscopy. Application to cyclic nucleotides. J. Magn. Reson. 39, 399–412 (1980).

[57] R. Baumann, G. Wider, R. R. Ernst, K. Wüjthrich, Improvement of 2D NOE and 2D correlated spectra by symmetrization. J. Magn. Reson. 44, 402–406 (1981).

[58] G. A. Morris, Compensation of instrumental imperfections by deconvolution using an internal reference signal. J. Magn. Reson. 80, 547–552 (1988).

[59] A. Gibbs, G. A. Morris, Reference deconvolution:, Elimination of distortions arising from reference line truncation. J. Magn. Reson. 91, 77–83 (1991).

[60] A. Gibbs, G. A. Morris, A. G. Swanson, D. Cowburn, Suppression of *t*_1_ noise in 2D NMR spectroscopy by reference deconvolution. J. Magn. Reson. 101, 351–356 (1993).

[61] C. Brissac, T. E. Malliavin, M. A. Delsuc, Use of the cadzow procedure in 2D NMR for the reduction of *t*_1_ noise. J. Biomol. NMR 6, 361–365 (1995). 22910877 10.1007/BF00197635

[62] S. Poulding, A. J. Charlton, J. Donarski, J. C. Wilson, Removal of *t*_1_ noise from metabolomic 2D ^1^H-^13^C HSQC NMR spectra by correlated trace denoising. J. Magn. Reson. 189, 190–199 (2007). 17920317 10.1016/j.jmr.2007.09.004

[63] L. Song, J. Wang, X. Su, X. Zhang, C. Li, X. Zhou, D. Yang, B. Jiang, M. Liu, REAL-*t*_1_, an effective approach for *t*_1_-noise suppression in NMR spectroscopy based on resampling algorithm. Chin. J. Chem. 38, 77–81 (2020).

[64] D. Koprivica, R. P. Martinho, M. Novakovic, M. J. Jaroszewicz, L. Frydman, A denoising method for multidimensional magnetic resonance spectroscopy and imaging based on compressed sensing. J. Magn. Reson. 338, 107187 (2022). 35292421 10.1016/j.jmr.2022.107187

[65] S. Wei, Y. Ding, K. Song, Z. Liu, A robust *t*_1_ noise suppression method in NMR spectroscopy. Magn. Reson. Chem. 61, 473–480 (2023). 37143296 10.1002/mrc.5355

[66] P. J. Bowyer, A. G. Swanson, G. A. Morris, Randomized acquisition for the suppression of systematic *F*_1_ artifacts in two-dimensional NMR spectroscopy. J. Magn. Reson. 140, 513–515 (1999). 10497061 10.1006/jmre.1999.1882

[67] H. Mo, J. S. Harwood, D. Yang, C. B. Post, A simple method for NMR *t*_1_ noise suppression. J. Magn. Reson. 276, 43–50 (2017). 28103498 10.1016/j.jmr.2016.12.014 PMC5336490

[68] Y. Nishiyama, V. Agarwal, R. Zhang, *t*_1_-noise suppression by γ-free recoupling sequences in solid-state NMR for structural characterization of fully protonated molecules at fast MAS. J. Phys. Chem. C 124, 26332–26343 (2020).

[69] A. Venkatesh, X. Luan, F. A. Perras, I. Hung, W. Huang, A. J. Rossini, *t*_1_-noise eliminated dipolar heteronuclear multiple-quantum coherence solid-state NMR spectroscopy. Phys. Chem. Chem. Phys. 22, 20815–20828 (2020). 32914158 10.1039/d0cp03511d

[70] F. A. Perras, T. W. Goh, W. Huang, *t*_1_-noise elimination by continuous chemical shift anisotropy refocusing. Solid State Nucl. Magn. Reson. 120, 101807 (2022). 35709566 10.1016/j.ssnmr.2022.101807

[71] A. J. Robertson, M. K. Pandey, A. Marsh, Y. Nishiyama, S. P. Brown, The use of a selective saturation pulse to suppress *t*_1_ noise in two-dimensional ^1^H fast magic angle spinning solid-state NMR spectroscopy. J. Magn. Reson. 260, 89–97 (2015). 26432398 10.1016/j.jmr.2015.09.005

[72] Y. Ishii, J. P. Yesinowski, R. Tycko, Sensitivity enhancement in solid-state ^13^C NMR of synthetic polymers and biopolymers by ^1^H NMR detection with high-speed magic angle spinning. J. Am. Chem. Soc. 123, 2921–2922 (2001). 11456995 10.1021/ja015505j

[73] M. Shen, S. Wegner, J. Trébosc, B. Hu, O. Lafon, J. P. Amoureux, Minimizing the *t*_1_-noise when using an indirect ^1^H high-resolution detection of unlabeled samples. Solid State Nucl. Magn. Reson. 87, 111–116 (2017). 28688541 10.1016/j.ssnmr.2017.06.008

[74] F. A. Perras, M. Pruski, Reducing *t*_1_ noise through rapid scanning. J. Magn. Reson. 298, 31–34 (2019). 30513456 10.1016/j.jmr.2018.11.008

[75] T. J. Horne, G. A. Morris, P-Type gradient-enhanced COSY experiments show lower *t*_1_ noise than N-type. Magn. Reson. Chem. 35, 680–686 (1997).

[76] W. F. Reynolds, R. G. Enriquez, Gradient-selected versus phase-cycled HMBC and HSQC: Pros and cons. Magn. Reson. Chem. 39, 531–538 (2001).

[77] W. E. Maas, F. H. Laukien, D. G. Cory, Gradient, high resolution, magic angle sample spinning NMR. J. Am. Chem. Soc. 118, 13085–13086 (1996).

[78] T. M. Alam, J. E. Jenkins, “Spectroscopy is the study of absorption and emission of electromagnetic radiation due to the interaction between matter and energy,” in *Advanced Aspects of Spectroscopy* (Intech, 2012), pp. 279–306.

[79] C. A. Fyfe, J. Skibsted, H. Grondey, H. Meyer zu Altenschildesche, Pulsed field gradient multiple-quantum MAS NMR spectroscopy of half-integer spin quadrupolar nuclei. Chem. Phys. Lett. 281, 44–48 (1997).

[80] O. W. Sørensen, M. Rance, R. R. Ernst, *z* filters for purging phase- or multiplet-distorted spectra. J. Magn. Reson. 56, 527–534 (1984).

[81] J. G. Pelton, D. E. Wemmer, Heteronuclear NMR pulse sequences applied to biomolecules. Annu. Rev. Phys. Chem. 46, 139–168 (1995). 7495481 10.1146/annurev.pc.46.100195.001035

[82] U. R. Prabhu, S. R. Chaudhari, N. Suryaprakash, Visualization of enantiomers and determination of homo- and hetero-nuclear residual dipolar and scalar couplings: The natural abundant ^13^C edited J/D-resolved NMR techniques. Chem. Phys. Lett. 500, 334–341 (2010).

[83] Ē. Kupče, T. D. W. Claridge, Molecular structure from a single NMR supersequence. Chem. Commun. 54, 7139–7142 (2018). 10.1039/c8cc03296c 29855011

[84] Ē. Kupče, T. D. W. Claridge, New NOAH modules for structure elucidation at natural isotopic abundance. J. Magn. Reson. 307, 106568 (2019). 31421539 10.1016/j.jmr.2019.106568

[85] A. Lesage, C. Auger, S. Caldarelli, L. Emsley, Determination of through-bond carbon-carbon connectivities in solid-state NMR using the INADEQUATE experiment. J. Am. Chem. Soc. 119, 7867–7868 (1997).

[86] A. Lesage, M. Bardet, L. Emsley, Through-bond carbon-carbon connectivities in disordered solids by NMR. J. Am. Chem. Soc. 121, 10987–10993 (1999).

[87] S. Cadars, J. Sein, L. Duma, A. Lesage, T. N. Pham, J. H. Baltisberger, S. P. Brown, L. Emsley, The refocused INADEQUATE MAS NMR experiment in multiple spin-systems: Interpreting observed correlation peaks and optimising lineshapes. J. Magn. Reson. 188, 24–34 (2007). 17588789 10.1016/j.jmr.2007.05.016

[88] K. Takegoshi, S. Nakamura, T. Terao, ^13^C–^1^H dipolar-assisted rotational resonance in magic-angle spinning NMR. Chem. Phys. Lett. 344, 631–637 (2001).

[89] G. De Paëpe, Dipolar recoupling in magic angle spinning solid-state nuclear magnetic resonance. Annu. Rev. Phys. Chem. 63, 661–684 (2012). 22404583 10.1146/annurev-physchem-032511-143726

[90] A. Brinkmann, M. Edén, M. H. Levitt, Synchronous helical pulse sequences in magic-angle spinning nuclear magnetic resonance: Double quantum recoupling of multiple-spin systems. J. Chem. Phys. 112, 8539–8554 (2000).

[91] M. Carravetta, M. Edén, X. Zhao, A. Brinkmann, M. H. Levitt, Symmetry principles for the design of radiofrequency pulse sequences in the nuclear magnetic resonance of rotating solids. Chem. Phys. Lett. 321, 205–215 (2000).

[92] A. Brinkmann, M. H. Levitt, Symmetry principles in the nuclear magnetic resonance of spinning solids: Heteronuclear recoupling by generalized Hartmann-Hahn sequences. J. Chem. Phys. 115, 357–384 (2001).

[93] G. Pileio, M. Concistrè, N. McLean, A. Gansmüller, R. C. D. Brown, M. H. Levitt, Analytical theory of γ-encoded double-quantum recoupling sequences in solid-state nuclear magnetic resonance. J. Magn. Reson. 186, 65–74 (2007). 17303455 10.1016/j.jmr.2007.01.009

[94] K. Saalwächter, F. Lange, K. Matyjaszewski, C.-F. Huang, R. Graf, BaBa-xy16: Robust and broadband homonuclear DQ recoupling for applications in rigid and soft solids up to the highest MAS frequencies. J. Magn. Reson. 212, 204–215 (2011). 21803622 10.1016/j.jmr.2011.07.001

[95] P. E. Kristiansen, M. Carravetta, J. D. van Beek, W. C. Lai, M. H. Levitt, Theory and applications of supercycled symmetry-based recoupling sequences in solid-state nuclear magnetic resonance. J. Chem. Phys. 124, 234510 (2006). 16821932 10.1063/1.2205857

[96] Z. Zhang, H. Liu, J. Deng, R. Tycko, J. Yang, Optimization of band-selective homonuclear dipolar recoupling in solid-state NMR by a numerical phase search. J. Chem. Phys. 150, 154201 (2019). 31005077 10.1063/1.5092986 PMC6474779

[97] P. E. Kristiansen, M. Carravetta, W. C. Lai, M. H. Levitt, A robust pulse sequence for the determination of small homonuclear dipolar couplings in magic-angle spinning NMR. Chem. Phys. Lett. 390, 1–7 (2004).

[98] K. Märker, S. Hediger, G. De Paëpe, Efficient 2D double-quantum solid-state NMR spectroscopy with large spectral widths. Chem. Commun. 53, 9155–9158 (2017). 10.1039/c7cc04890d 28765850

[99] M. Bak, J. T. Rasmussen, N. C. Nielsen, SIMPSON: A general simulation program for solid-state NMR spectroscopy. J. Magn. Reson. 213, 366–400 (2011). 22152357 10.1016/j.jmr.2011.09.008

[100] D. W. Juhl, Z. Tošner, T. Vosegaard, “Versatile NMR simulations using SIMPSON,” in *Annual Reports on NMR Spectroscopy* (Elsevier, 2020), vol. 100, pp. 1–59.

[101] M. O. Boles, R. J. Girven, The structures of ampicillin: A comparison of the anhydrate and trihydrate forms. Acta Crystallogr. B Struct. Crystallogr. Cryst. Chem. 32, 2279–2284 (1976).

[102] E. Bouleau, P. Saint-Bonnet, F. Mentink-Vigier, H. Takahashi, J.-F. Jacquot, M. Bardet, F. Aussenac, A. Purea, F. Engelke, S. Hediger, D. Lee, G. De Paëpe, Pushing NMR sensitivity limits using dynamic nuclear polarization with closed-loop cryogenic helium sample spinning. Chem. Sci. 6, 6806–6812 (2015). 28757972 10.1039/c5sc02819a PMC5508678

[103] D. Marion, M. Ikura, R. Tschudin, A. Bax, Rapid recording of 2D NMR spectra without phase cycling. Application to the study of hydrogen exchange in proteins. J. Magn. Reson. 85, 393–399 (1989).

[104] R. S. Thakur, N. D. Kurur, P. K. Madhu, Swept-frequency two-pulse phase modulation for heteronuclear dipolar decoupling in solid-state NMR. Chem. Phys. Lett. 426, 459–463 (2006).

[105] R. S. Thakur, N. D. Kurur, P. K. Madhu, Improved heteronuclear dipolar decoupling sequences for liquid-crystal NMR. J. Magn. Reson. 185, 264–269 (2007). 17257867 10.1016/j.jmr.2007.01.003

[106] M. Leskes, R. S. Thakur, P. K. Madhu, N. D. Kurur, S. Vega, Bimodal Floquet description of heteronuclear dipolar decoupling in solid-state nuclear magnetic resonance. J. Chem. Phys. 127, 024501 (2007). 17640131 10.1063/1.2746039

[107] W. I. Goldburg, M. Lee, Nuclear magnetic resonance line narrowing by a rotating rf field. Phys. Rev. Lett. 11, 255–258 (1963).

