# Supplementary material for: Artifact-free ultralow-temperature DNP-enhanced NMR of molecular assemblies at natural isotopic abundance
Source: Sci Adv. 2025 Dec 19;11(51):eaeb0337. doi: 10.1126/sciadv.aeb0337 (PMC12716396; doi:10.1126/sciadv.aeb0337)
Supplement: Supplementary file 1 — Sections S1 to S8 Figs. S1 to S7 References [file sciadv.aeb0337_sm.pdf]

Science Advances
AAAS

Supplementary Materials for

Artifact-free ultralow-temperature DNP-enhanced NMR of molecular assemblies at natural isotopic abundance

Quentin Reynard-Feytis *et al.*

Corresponding author: Gaël De Paëpe, gael.depaepe@cea.fr

Sci. Adv. **11**, eaeb0337 (2025)
DOI: 10.1126/sciadv.aeb0337

This PDF file includes:

Sections S1 to S8
Figs. S1 to S7
References

Supplementary Text

S1 Practical aspects of the *zz*-filter implementation

This section provides the practical details required for the implementation and optimization of the *zz*-filter, including the corresponding phase cycling scheme. We illustrate the procedure with a generic dipolar recoupling sequence, programmed for both 1D and 2D DQ/SQ experiments using the `mc` command.

Implementation of the zz-filter sequence

At the beginning of the sequence, the parameters of the *z*- and *zz*-filters must be defined. In particular, the *zz*-filter delay `d30` is calculated from the rotor period `d31` and the number of rotor cycles `l30`:

```
;d3 : z-filter delay before detection (e.g. 3 ms)
;l30 : number of rotor periods for zz-filter
;d30 : zz-filter delay
;cpdprg4 : DARR field on 1H during zz-filter (cw, pl14)
;pl14 : 1H field strength during zz-filter

"d31=1s/cnst31"                 ; rotor period
#ifdef zzfilter
    "d30=d31*(l30-1) + d31-2*p1-2u" ; zz-filter delay
#endif
```

The sequence starts from the pointer `3` with an optional presaturation, the recycle delay `d1`, a ^{1}H→X CP transfer, and a flip-back pulse to store the magnetization along the *z*-axis. The DQ-excitation block follows, implemented at power level `pl21` with pulses of length `p21` and its corresponding phases (`ph21` and `ph22` in this example).

```
3 ... ; presat, recycle delay d1, cp, flip back, etc...
```

```
;====================== DQ-excitation =======================
1u pl21:f1 cpds3:f2 ; set X-channel recoupling power, 1H cw decoupling on


11 p21*4:f1 ph21
   p21*3:f1 ph22
        ...
        ...
lo to 11 times l0
```

After excitation, the zz-filter is applied if the option `-Dzz-filter` is enabled:

```
;========================== DQz-filter ==========================
#ifdef zzfilter


 1u pl11:f1 do:f2    ; set pulse power on X channel, turn off 1H decoupling


 p1:f1 ph5:r         ; zz-filter flip-up pulse, create IzSz terms
 d30 ;cpds4:f2       ; apply the zz-filter delay (optionnal DARR-field on 1H channel)
 p1:f1 ph5:r         ; zz-filter flip-back pulse, restore DQCs


 1u pl21:f1 cpds3:f2 ; set recoupling power on X channel, turn on 1H decoupling
#else
 1u
#endif
```

In this implementation, the zz-filter is rotor-synchronous. The 90° flip-up/down pulses use the phase list `ph5:r`, where an adjustable offset parameter `phcor5` allows easy optimization of the zz-filter phase, particularly when the phase of the DQ Hamiltonian is not known a priori. Optionally, a DARR-field (`cpds4`) can be applied on the ^{1}H channel during the zz-filter delay to accelerate the decay of undesired single-quantum coherences. Following the zz-filter, the sequence continues with t_1 evolution and DQ reconversion:

```
;========================== t1-evolution ==========================
```

```
0.3u cpds2:f2
d0
0.3u cpds3:f2

;======================== DQ-reconversion ========================
15 p21*4:f1 ph23        ; repeat l0 recoupling block (arbitrary example)
   p21*3:f1 ph24
        ...
        ...
 lo to 15 times l0
```

At this stage, part of the DQCs are reconverted into longitudinal magnetization. A short z-filter is then applied before detection:

```
;======================== reading pulse =========================
1u do:f2                ; turn off the decoupling before z-filter

d3                      ; apply z-filter before detection

(p1 ph29 pl11):f1       ;reading pulse
1u cpds2:f2             ; turn on decoupling before detection

;============================ Detection =========================
go=3 ph31
1m do:f2     ;1H decoupler off
```

Finally, the `mc` command is used to extend the 1D experiment to 2D DQ/SQ acquisition:

```
;save data in current data set
100m mc #0 to 3 F1PH(ip21 & ip22 & ip5, id0)

1m do:f2
```

```
HaltAcqu, 1m    ;Protection Halt
exit
```

Phase cycling of the zz-filter

In this example, the DQC → I_z transfer pathway is selected using a 4-step phase cycling on the reconversion block (ph23, ph24), as shown in the phase program below. In this case, the zz-filter doesn't need to be phase-cycled together with the reconversion block, as the phase of the DQCs remains the same.

```
;                        == DQ excitation pulses ==
ph21 = (8) 2
ph22 = (8) 6

;                        == DQ reconversion pulses ==
ph23 = (8) 2 4 6 0
ph24 = (8) 6 0 2 4

;                        == zz-filter pulses ==
ph5 = (8) 1              ; no phase cycling on the zz-filter

;                        == Receiver phase ==
ph29 = 0                 ; reading pulse phase
ph31 = 0 2 0 2           ; DQ selection phase cycling
```

On the other hand, when the DQ selection is performed during the excitation block, it becomes necessary to phase-cycle the zz-filter pulses together with the DQ-excitation pulses using the phases `ph5 = (8) 1 3 5 7`. Finally, it is worth noting that alternative implementations are possible — for instance, applying the zz-filter after the t_1 evolution — depending on the experimental context, though each configuration requires specific considerations.

Quadrature detection

The phase of the zz-filter must also be considered in relation to quadrature detection in the indirect dimension. In this work, we used States-TPPI, incrementing the excitation phases (`ph21`, `ph22`) by 45° (corresponding to a 90° shift in the DQ subspace, which is why we declared the DQ and zz-filter phases in a base of `(8)`). Since this modifies the phase of the generated DQCs, the zz-filter phase must be incremented simultaneously. This is implemented as:

```
100m mc #0 to 3 F1PH(ip21 & ip22 & ip5, id0)
```

Omitting this adjustment suppresses the imaginary component of the indirect dimension, producing a symmetric spectrum in the indirect dimension.

Rotor-synchronous vs. non-rotor-synchronous filters

In the present example, the zz-filter is rotor-synchronous. Whether rotor synchronization is required depends on the properties of the recoupling Hamiltonian:

- γ-free Hamiltonians (i.e., independent of the γ-angle): in this case, the zz-filter length can be arbitrary, since the excitation and reconversion Hamiltonians are identical regardless of the rotor phase. Examples include J-based sequences such as INADEQUATE (*54, 86*) and heteronuclear dipolar recoupling sequences TEDOR (*51*).

- γ-encoded Hamiltonians (i.e., only the Hamiltonian phase depends on the γ-angle): here, the zz-filter does not need to be strictly rotor-synchronous. However, the zz-filter delay must be considered when calculating the phase shift of the reconversion block to ensure proper matching with the excitation Hamiltonian despite the t_1-evolution. Although not mandatory, keeping the zz-filter rotor-synchronous greatly simplifies its implementation. Typical examples include most symmetry-based sequences (*93*).

- Non-γ-encoded Hamiltonians (i.e., both amplitude and phase depend on the γ-angle): in this situation, rotor synchronization of the zz-filter is generally required to preserve the correspondence between excitation and reconversion Hamiltonians.

Overall, while using a rotor-synchronous zz-filter is not always necessary, it considerably simplifies its systematic use and does not come with any penalty. Therefore, we decided to keep the zz-filter rotor-synchronous.

Optimization of the zz-filter

The optimization of the zz-filter involves three main aspects: phase, delay, and optional DARR-field.

1. **Phase optimization**: The first step is to ensure that the filter phase maximizes the DQC $\rightarrow I_{jz}I_{kz}$ conversion. In principle, the zz-filter phases can be deduced from the phase of the DQ Hamiltonian, but this is not always straightforward in practice. A practical approach is to optimize the `ph5` offset parameter `phcor5` (in degrees) within the range -90° to +90°. This allows one to verify that the phases are correctly adjusted for the specific DQ Hamiltonian in use.

2. **Delay optimization**: The second parameter is the minimal filter delay `d30`, which must be long enough to ensure efficient dephasing of residual SQCs. A convenient way to determine this is to record a single-scan experiment while varying the number of rotor periods `l30`. Without phase cycling, the spectrum is dominated by SQCs, which are progressively dephased during the zz-filter. This procedure quickly indicates the minimal delay required for effective suppression. It should be noted, however, that incomplete SQC suppression can still be compensated by the final z-filter before detection or by the phase cycling, so the zz-filter may remain effective even if SQCs are not fully dephased.

3. **Optional DARR-field irradiation**: A final degree of freedom is the application of a DARR field on the ^{1}H channel during the zz-filter delay. This is implemented by applying continuous-wave irradiation with a dedicated power level (e.g., `pl14`) during the zz-filter delay `d30` (enabled by uncommenting the `cpds4` command in the sequence). To determine the optimal condition, the zz-filter delay can be set to $\simeq$ 1 ms, and the irradiation amplitude `pl14` optimized in a single-scan experiment. Since such experiments are dominated by SQCs, the signal reaches a minimum when the irradiation satisfies the DARR matching condition $\omega_{^1H} = n\omega_r$ with $n = 1, 2$). This minimum thus provides a direct criterion for setting the optimal DARR-field strength.

S2 Application of the zz-filter to the heteronuclear TEDOR experiment

The combination of z- and zz-filter has already been implemented in the zfr-TEDOR by Jaroniec and coworkers (*51*) in the context of ^{13}C,^{15}N-labelled molecules. However, their purpose was not to address t_1-noise, but to suppress MQCs and antiphase terms generated by the J-coupling, which lead to spurious cross-peaks and line shape distortions. The zz-filter occurs after the first REDOR block, which creates antiphase coherences such as

$$I_x \xrightarrow{\mathcal{H}_{\text{REDOR}} \propto 2I_zS_z} \cos\left(\frac{\omega t_{\text{mix}}}{2}\right) I_x + \sin\left(\frac{\omega t_{\text{mix}}}{2}\right) 2I_yS_z \tag{S1}$$

where the I_i and S_i operators, with $i \in \{x,\ y,\ z\}$, correspond respectively to ^{13}C and ^{15}N nuclei. In this case, the design and implementation of the zz-filter is straightforward, as it just required to set accordingly the delay Δ_{zz} between the $(\pi/2)_{x,I}$ and the $(\pi/2)_{x,S}$ pulses (see Figure S1A). The first pulse converts the antiphase $2I_yS_z$ into zz-terms, which pass the zz-filter, while uncoupled spins components remain along x and are dephased. The detailed sequence with pulse phases is described in ref (*51*) (**B**) 2D ^{15}N-^{13}C TEDOR spectra recorded on natural abundance ampicillin with 3 ms z-filter only (i) and with both z- and zz-filter of 3 ms (ii). The contour levels are normalized to the maximum intensity in each spectrum. At natural abundance, unwanted MQCs that cause problems are negligible. As shown in Figure S1B, the zz-filter originally implemented to remove MQCs artefacts also efficiently reduce t_1-noise in experiments at natural abundance.

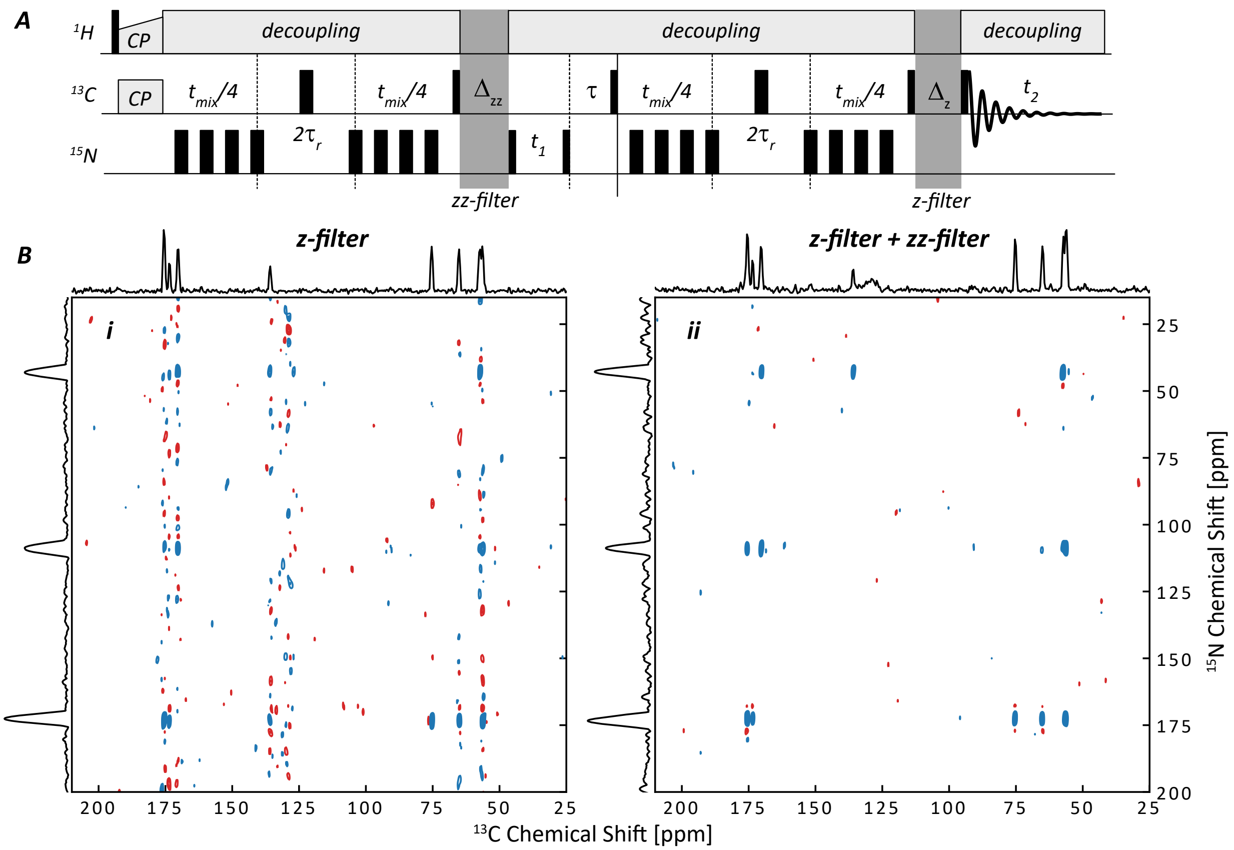

Figure S1: Impact of the zz-filter on the t_1-noise in DNP-enhanced zfr-TEDOR experiments. (**A**) 2D ^{13}C,^{15}N zfr-TEDOR sequence. π and $\pi/2$ pulses are represented by narrow and wide black rectangles, respectively, t_{mix} corresponds to the TEDOR mixing time and the z- and zz-filter delays are Δ_z and Δ_{zz}, respectively.

S3 Noise analysis

We show in Figure S2 the f_1-traces used to determine the $\sigma_{\mathrm{t1}}/\sigma_{\mathrm{th}}$ ratio, the zz-filter efficiency η_{zz}, and the signal-to-noise ratio (SNR) along f_1. The signal-free regions used to calculate the $t1$-noise standard deviation σ_{t1} are highlighted in red. These values are reported in Figure Figure S2 for: (A) the refocused INADEQUATE spectrum (Figure 2), (B) the SR26 dipolar-recoupling experiment at 100 K (Figure 4), and (C) the SR26 dipolar-recoupling experiment at 30 K (Figure 5). For comparison, an additional f_1-trace with lower t_1-noise from the refocused INADEQUATE experiment (Figure S2B.ii) is also included to illustrate the effect of the zz-filter on traces with less $t1$-noise.

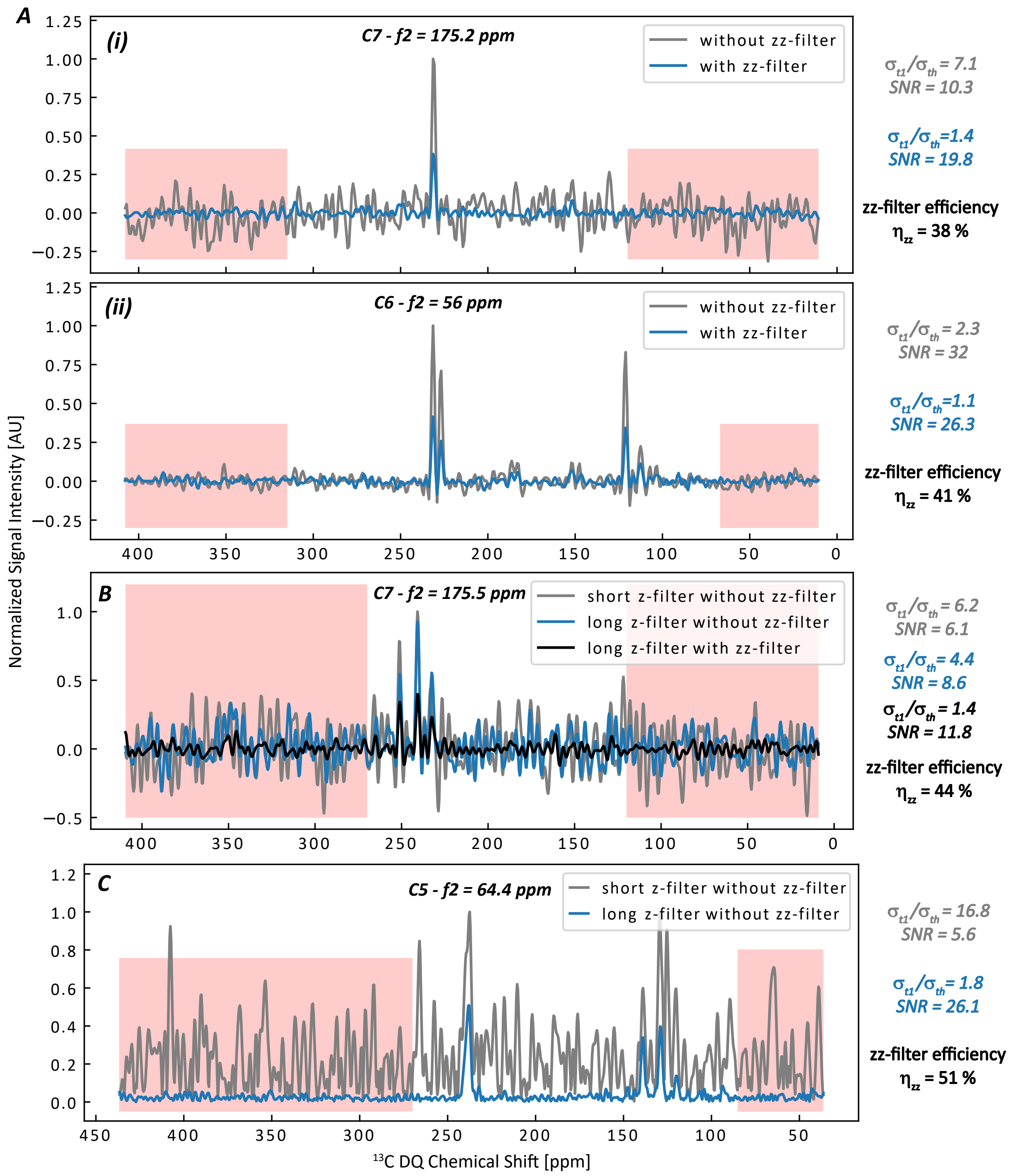

Figure S2: Details on the f_1-traces analysis for σ_{t1}, SNR, and η_{zz} in refocused-INADEQUATE and SR26 experiments at 100 K and 30 K. Representation of the f_1-traces with the detail of the σ_{t1} measurements for the calculation of the σ_{t1}/σ_{th} ratio as well as the SNR and the zz-filter efficiency η_{zz} in the case of the refocused INADEQUATE corresponding to the Figure 2 (**A**), the dipolar-recoupling SR26 sequence at 100 K corresponding to the Figure 4 (**B**) and at 30K corresponding to the Figure 5 (**C**). Each f_1-trace is represented and normalized to the trace of the spectrum without zz-filter and with a long z-filter (3 ms). The red shaded regions represent the part of the f_1-traces which is used to calculate the noise standard deviation.

S4 Additional spectra for the zz-filtered refocused-INADEQUATE

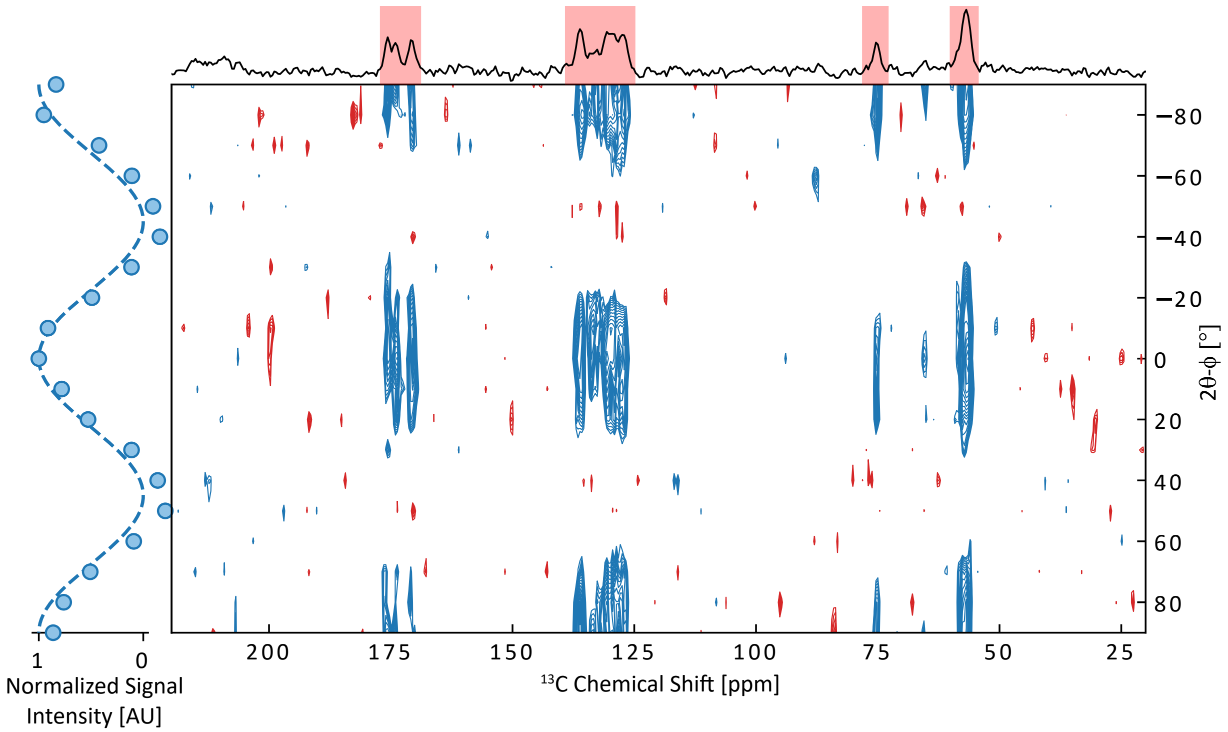

Figure S3: zz-filter efficiency dependence on $2\theta - \phi$ in refocused-INADEQUATE. DQ-filtered normalized signal of refocused-INADEQUATE with zz-filter with respect to $2\theta - \phi$, θ being the zz-filter 90° pulses phase and ϕ the phase of the generated DQCs (i.e., 90° in this case). The black line shows the projection among the horizontal axis, red squares indicate the region selected to integration and calculate the normalized signal intensity for each $2\theta - \phi$ value. The intensity is represented with the blue dots on the left and normalized to the initial points $2\theta - \phi$=0°. The blue dashed line corresponds to the theoretical value $\cos{(2\theta - \phi)^2}$.

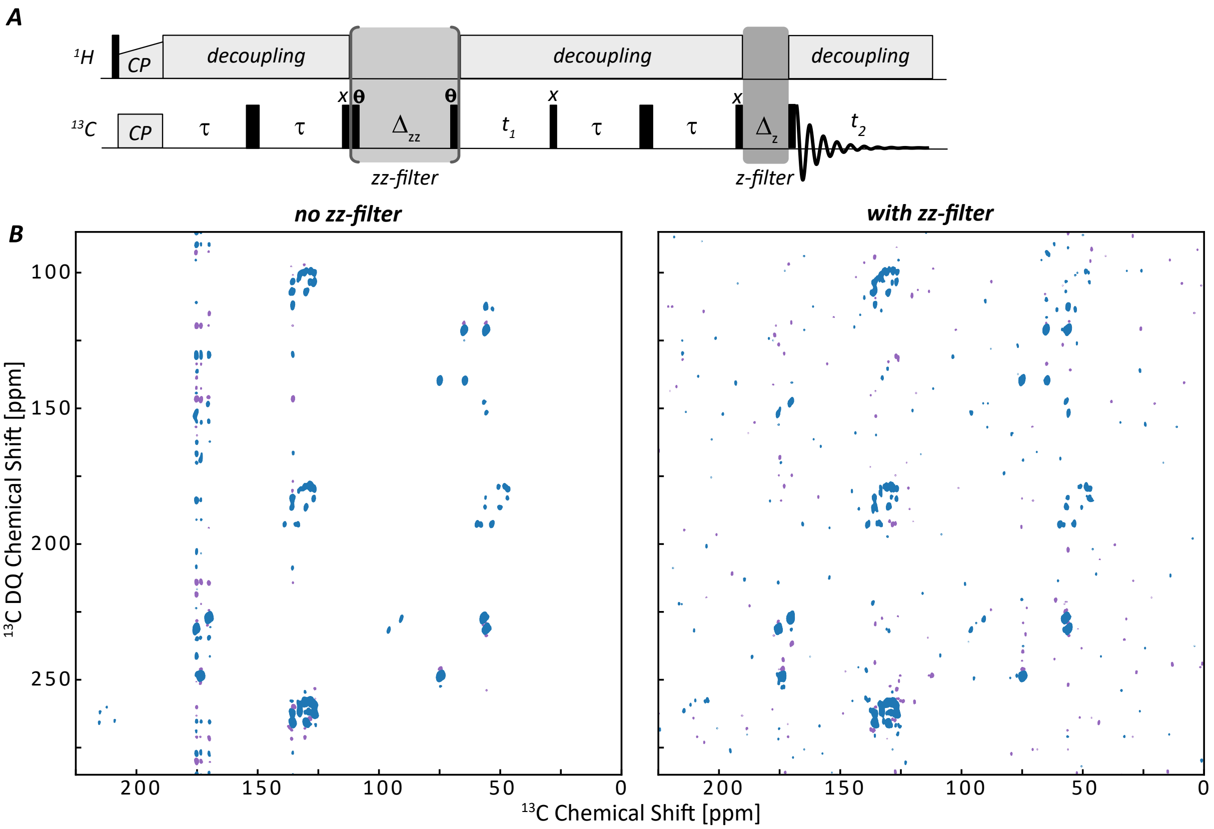

Figure S4: Refocused-INADEQUATE sequences and full spectra. (**A**) z- and zz-filtered J-refocused-INADEQUATE pulse sequence. π and $\pi/2$ pulses are represented by narrow and wide black rectangles, respectively, and the z- and zz-filter delays are Δ_z and Δ_{zz}, respectively. (**B**) DQ/SQ refocused-INADEQUATE DNP-enhanced spectra on natural abundance ampicillin microcrystals impregnated with cAsymPol-POK, without (on the left) and with (on the right) a zz-filter of 6.25 ms (standard z-filter set to 3 ms). Contour levels are normalized to the maximum intensity in each spectrum.

S5 Accelerating dephasing of SQCs from isolated spins by adding a DARR-field during the zz-filter

In the scope of this study, we showed the implementation of the zz-filter to suppress t_1-noise in relatively low MAS spinning speeds (<10kHz), on a model system with abundant ^{1}H. Under such conditions, ^{1}H-^{13}C and ^{1}H-^{1}H dipolar as well as ^{13}C largest CSA (for aromatics and carbonyls) interactions are not completely averaged out by MAS, which enables efficient dephasing of transverse coherences during the zz-filter. Depending on the ^{13}C chemical site, SQCs are more or less efficiently dephased, and we observed that the dephasing of carbonyl ^{13}C takes more time than of aliphatics and therefore requires a longer zz-filter delay (Figure S5A). For experiments at higher spinning frequencies or samples with less ^{1}H, the introduction of a DARR field (*88*) on the ^{1}H

channel during the zz-filter delay, which satisfies the conditions $\omega_{1H} = n\omega_r$ with $n = 1$ or 2, can then help for a faster dephasing, as shown in Figure S5B. Both conditions reintroduce both the CSA and ^{1}H-^{13}C heteronuclear dipolar interactions, while the first condition ($n = 1$) also reintroduces ^{1}H-^{1}H homonuclear dipolar coupling. While the signals from COs are not completely dephased after ≃ 3 ms of zz-filter without irradiation (Figure S5A.i), they are completely dephased after only ≃ 1 ms of zz-filter with the application of a DARR-field on the ^{1}H channel (Figure S5B.i). The use of the DARR field during the zz-filter has no impact on the DQ signal, which successfully pass through the zz-filter (Figure S5A.ii vs Figure S5B.ii). In terms of t_1-noise suppression, the DARR-field was not mandatory in the regime we tested it, but might become crucial in other experimental conditions, especially at high-spinning frequencies.

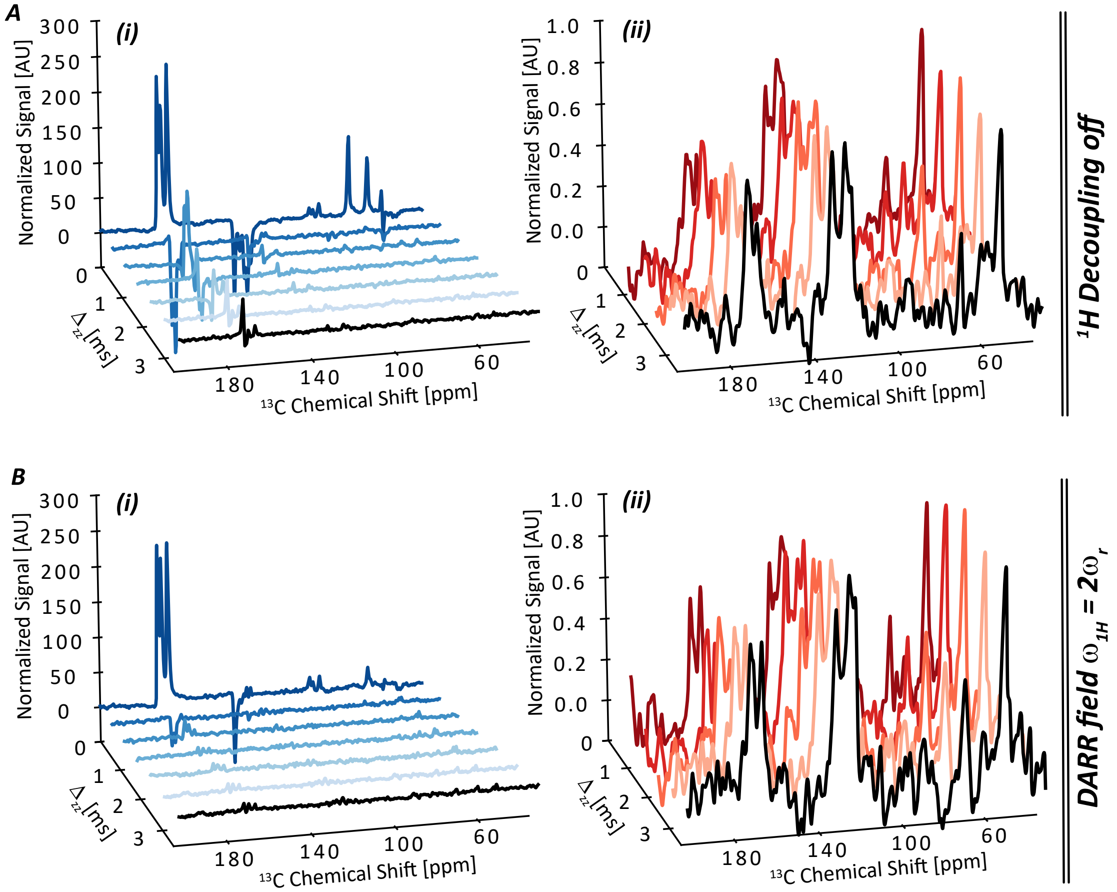

Figure S5: DARR field accelerates single-quantum coherence dephasing. (**A**) 1D signal detected in a J-refocused INADEQUATE experiment as a function of the zz-filter duration Δ_{zz}, with $t_1 = 0$: (i) single-scan signal showing the dephasing of SQCs, and (ii) phase-cycled signal of 64 scans data showing conserved DQ-filtered signal intensity during Δ_{zz}. (**B**) Same as (A) but with a DARR field ($\omega_{1H} = 2\omega_r$) of 16 kHz applied on the ^{1}H channel during the zz-filter Δ_{zz}.

S6 Impact of the z-filter on the t_1-noise level for the refocused-INADEQUATE

In the main text, we discussed the importance of applying a z-filter prior to detection in dipolar-based polarization transfer experiments to suppress residual t_1-noise which remains when using only a zz-filter. We also noted that we didn't face this issue in J-based refocused INADEQUATE experiment, where a z-filter is almost systematically applied prior to detection, as originally proposed by Cadars and co-workers (*87*), to dephase multiple-quantum coherences (MQCs) that lead to spurious peaks and peak distortions in labeled spin systems. Here, we investigate the impact of the z-filter on the t_1-noise level in refocused-INADEQUATE experiments. The results shown in Figure S6 were acquired under conditions similar to those in Figure 1 and Figure 2. The short and long z-filter delays correspond to 10 µs and 3 ms, respectively, and the zz-filter delay was set to approximately 3.1 ms when applied. A comparison of panels A and C of Figure S6 shows that, similar to the SR26 dipolar-based experiments, the application of a z-filter alone appears to reduce t_1-noise. However, repeating the same experiment as panel C later the same day yielded the spectrum in panel D, which displays significantly less t_1-noise, despite identical conditions. This suggests that the observed t_1-noise may be influenced by external factors whose stability varies over time. Consequently, the level of t_1-noise may fluctuate between otherwise equivalent experiments. Importantly, the combination of both zz-filter and z-filter consistently prevents t_1-noise contamination, offering a robust strategy for its suppression.

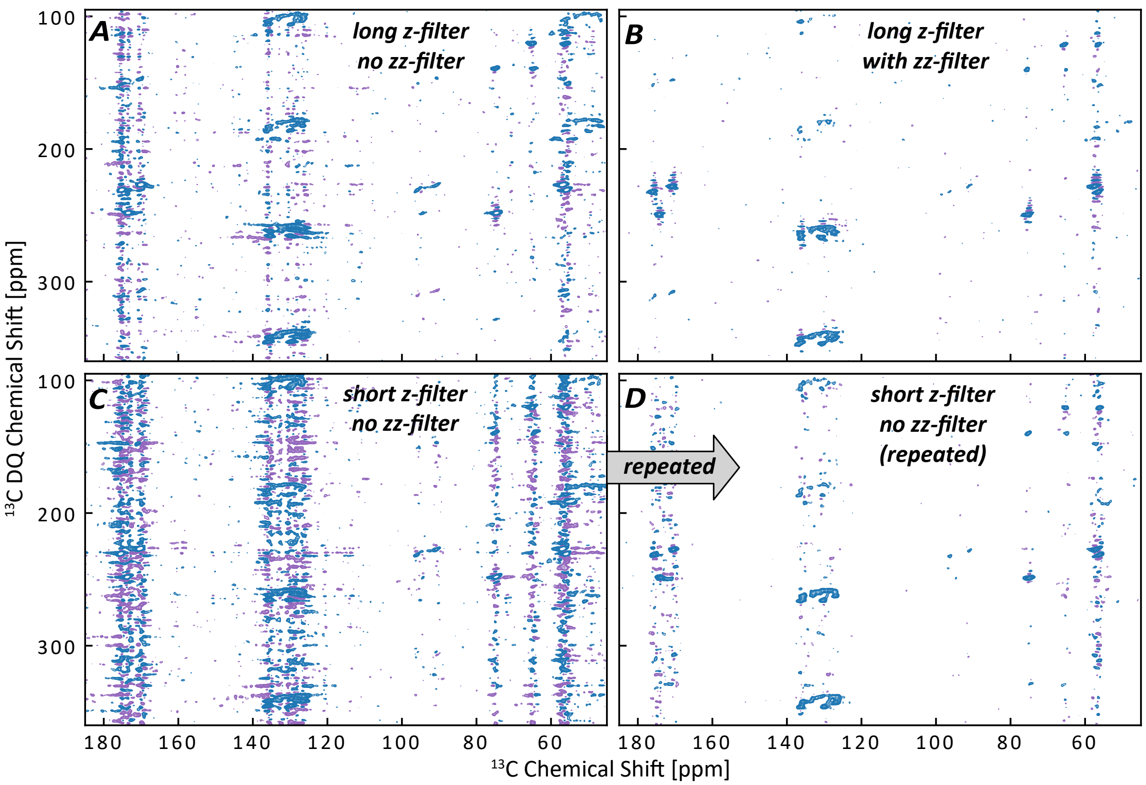

Figure S6: Impact of the z- and zz-filters on the t_1-noise in DNP-enhanced DQ/SQ refocused-INADEQUATE experiments. 2D DNP-enhanced DQ/SQ refocused-INADEQUATE spectra of natural abundance ampicillin (**A**) with a 3 ms long *z*-filter and no *zz*-filter, (**B**) with a 3 ms *zz*-filter in addition to the 3 ms long *z*-filter, (**C**) with a shorter *z*-filter of 100 µs and (**D**), in the same conditions than (C) but repeated later within the same day.

S7 zz-filter efficiency for γ-encoded/non-γ-encoded sequences

As explained in the main text, γ-sequences experience a higher signal loss ($\eta_{zz} = 0.25$ instead of 0.5) upon application of the zz-filter, due to the phase dependence of the DQCs with respect to the angle γ. To validate our theoretical predictions, we present in Figure S7A the DQ-filtered signals obtained for the SR26 and R26 sequences, both with and without application of a zz-filter. To ensure a meaningful comparison with the theoretical efficiencies, the spectra acquired with the zz-filter were normalized by the corresponding theoretical efficiency of each sequence. Both datasets were recorded under identical experimental conditions, on the 100 K Bruker MAS-DNP system described above, with a spinning speed of 6.5 kHz, a mixing time of 4.9 ms, and a zz-filter duration of 7.6 ms. In parallel, build-up simulations were performed using SIMPSON (*99, 100*) for both SR26 and R26, with and without the zz-filter, as shown in Figure S7B. The simulations used a dipolar coupling constant of 1.5 kHz, no chemical shift, the `rep144` crystal file with 40 γ-angles, and a spinning speed of 8 kHz. As illustrated in Figure S7B, the expected zz-filter efficiencies of 0.5 and 0.25 were verified for non-γ and γ-encoded sequences, respectively.

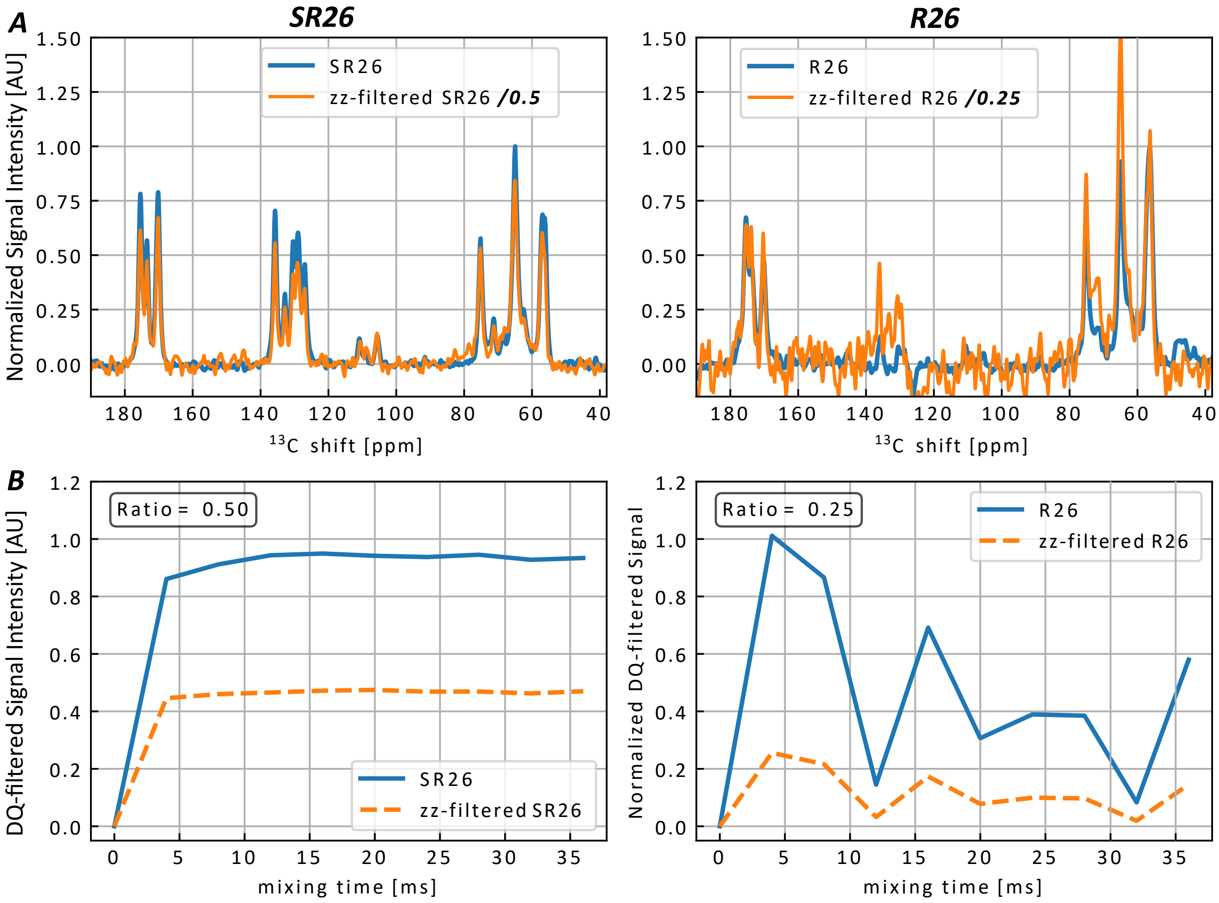

Figure S7: Comparison of the zz-filter efficiency for γ- vs non-γ-encoded sequences. (**A**) DQ-filtered signal for SR26 and R26 without zz-filter (blue curve) and with zz-filter (orange curve) with a mixing time of 4 ms. The spectra recorded with the use of the zz-filter are represented with their intensity divided by the theoretical efficiency of the zz-filter for both sequences (0.5 and 0.25 for SR26 and R26 respectively). (**B**) Build-up simulations of the zz-filter efficiency for SR26 (left panel) and R26 (right panel) with and without zz-filter (blue and orange dashed lines respectively).

S8 Derivation of Eq. (6)

Double quantum spectroscopy is based on the use of a sequence that introduces a double-quantum effective Hamiltonian which generates DQC noted as:

$$DQ_\phi = \cos(\phi) DQ_x + \sin(\phi) DQ_y \tag{S2}$$

with the DQ and ZQ operators in Eq. (S3) forming the DQ and ZQ subspaces respectively:

$$\begin{aligned} \mathrm{DQ}_x &= I_{jx}I_{kx} - I_{jy}I_{ky} = \tfrac{1}{2}(I_j^+I_k^+ + I_j^-I_k^-) \qquad & \mathrm{DQ}_y &= I_{jx}I_{ky} + I_{jy}I_{kx} = \tfrac{1}{2i}(I_j^+I_k^+ - I_j^-I_k^-) \\ \mathrm{ZQ}_x &= I_{jx}I_{kx} + I_{jy}I_{ky} = \tfrac{1}{2}(I_j^+I_k^- + I_j^-I_k^+) \qquad & \mathrm{ZQ}_y &= I_{jy}I_{kx} - I_{jx}I_{ky} = \tfrac{1}{2i}(I_j^+I_k^- - I_j^-I_k^+) \end{aligned} \tag{S3}$$

Let's suppose that we generate DQCs among DQ_x, we can study the way DQ_x is transformed by a generic $(\pi/2)_\theta$ pulse. For this, we can introduce the operators $I_{j\theta}$ and $I_{j\bar{\theta}}$, whose expression can be given by:

$$I_{j\theta} = c\theta I_{jx} + s\theta I_{jy} \qquad I_{j\bar{\theta}} = -s\theta I_{jx} + c\theta I_{jy} \tag{S4}$$

with the notation $s\alpha^n \equiv \sin(\alpha)^n$ and $c\alpha^n \equiv \cos(\alpha)^n$. Reciprocally, we can write:

$$I_{jx} = c\theta I_{j\theta} - s\theta I_{j\bar{\theta}} \qquad I_{jy} = s\theta I_{j\theta} + c\theta I_{j\bar{\theta}} \tag{S5}$$

One can note that the I_θ and $I_{\bar{\theta}}$ operators satisfy the same commutation property than I_x and I_y

$$[I_\theta, I_{\bar{\theta}}] = iI_z \tag{S6}$$

This allows us to calculate the effect of an arbitrary $(\pi/2)_\theta$ pulse

$$I_{jx} \xrightarrow{(\pi/2)_\theta} c\theta^2 I_{jx} + c\theta s\theta I_{jy} - s\theta I_{jz} \qquad I_{jy} \xrightarrow{(\pi/2)_\theta} s\theta c\theta I_{jx} + s\theta^2 I_{jy} + c\theta I_{jz} \tag{S7}$$

Now that these relations are established, we can calculate the effect of a general $(\pi/2)_\theta$ pulse on DQCs. A convenient way to tackle this problem is to calculate this on DQ_x, before generalizing it.

$$\begin{aligned} DQ_x = I_{jx}I_{kx} - I_{jy}I_{ky} \xrightarrow{(\pi/2)_\theta} & (c\theta^2 I_{jx} + c\theta s\theta I_{jy} - s\theta I_{jz})(c\theta^2 I_{kx} + c\theta s\theta I_{ky} - s\theta I_{kz}) \\ & -(c\theta s\theta I_{jx} + s\theta^2 I_{jy} + c\theta I_{jz})(c\theta s\theta I_{kx} + s\theta^2 I_{ky} + c\theta I_{kz}) \end{aligned} \tag{S8}$$

This can be developed and simplified using the notation $I_{\nu\mu} = I_{j\nu}I_{k\mu}$

$$\begin{aligned} &= (c\theta^4 I_{xx} + c\theta^3 s\theta I_{xy} - c\theta^2 s\theta I_{xz} + c\theta^3 s\theta I_{yx} + c\theta^2 s\theta^2 I_{yy} - c\theta s\theta^2 I_{yz} - c\theta^2 s\theta I_{zx} - c\theta s\theta^2 I_{zy} + s\theta^2 I_{zz}) \\ &\quad -(c\theta^2 s\theta^2 I_{xx} + c\theta s\theta^3 I_{xy} + c\theta^2 s\theta I_{xz} + c\theta s\theta^3 I_{yx} + s\theta^4 I_{yy} + c\theta s\theta^2 I_{yz} + c\theta^2 s\theta I_{zx} + c\theta s\theta^2 I_{zy} + c\theta^2 I_{zz}) \end{aligned} \tag{S9}$$

and we can rearrange the expression

$$
\begin{aligned}
=&(c\theta^2 - s\theta^2)\left[c\theta^2 I_{xx} + s\theta^2 I_{yy} + c\theta s\theta\overbrace{(I_{xy} + I_{yx})}^{DQ_y} - I_{zz}\right] - 2c\theta s\theta\left[\overbrace{c\theta I_{xz} + s\theta I_{yz}}^{I_{\theta z}} + \overbrace{c\theta I_{zx} + s\theta I_{zy}}^{I_{z\theta}}\right]\\
=&c(2\theta)\left[(1 - s\theta^2)I_{xx} + s\theta^2 I_{yy} + c\theta s\theta DQ_y - I_{zz}\right] - s(2\theta)\left[I_{\theta z} + I_{z\theta}\right]\\
=&c(2\theta)\Big[\overbrace{I_{xx}}^{\frac{1}{2}(DQ_x+ZQ_x)} -s\theta^2\overbrace{(I_{xx} - I_{yy})}^{DQ_x} + c\theta s\theta DQ_y - I_{zz}\Big] - s(2\theta)\left[I_{\theta z} + I_{z\theta}\right]\\
=&c(2\theta)\left[\frac{1}{2}ZQ_x + \frac{1}{2}(c\theta^2 - s\theta^2)DQ_x + \frac{1}{2}s(2\theta)DQ_y - I_{zz}\right] - s(2\theta)\left[I_{\theta z} + I_{z\theta}\right]\\
=&\frac{1}{2}c(2\theta)\Big[ZQ_x + \overbrace{c(2\theta)DQ_x + s(2\theta)DQ_y}^{DQ_{2\theta}} - 2I_{zz}\Big] - s(2\theta)\left[I_{\theta z} + I_{z\theta}\right]
\end{aligned}
\tag{S10}
$$

Finally, the final expression of the transformation of DQ_x under a pulse $(\pi/2)_\theta$ is given by

$$
DQ_x \xrightarrow{(\pi/2)_\theta} \frac{1}{2}c(2\theta)\left[ZQ_x + DQ_{2\theta} - 2I_{zz}\right] - s(2\theta)\left[I_{\theta z} + I_{z\theta}\right] \tag{S11}
$$

REFERENCES

1. E. R. Andrew, A. Bradbury, R. G. Eades, Nuclear magnetic resonance spectra from a crystal rotated at high speed. *Nature* **182**, 1659–1659 (1958).

2. I. J. Lowe, Free induction decays of rotating solids. *Phys. Rev. Lett.* **2**, 285–287 (1959).

3. K. Jaudzems, T. Polenova, G. Pintacuda, H. Oschkinat, A. Lesage, DNP NMR of biomolecular assemblies. *J. Struct. Biol.* **206**, 90–98 (2019).

4. A. G. M. Rankin, J. Trébosc, F. Pourpoint, J.-P. Amoureux, O. Lafon, Recent developments in MAS DNP-NMR of materials. *Solid State Nucl. Magn. Reson.* **101**, 116–143 (2019).

5. B. Reif, S. E. Ashbrook, L. Emsley, M. Hong, Solid-state NMR spectroscopy. *Nat. Rev. Methods Primers* **1**, 2 (2021).

6. C. P. Gordon, L. Lätsch, C. Copéret, Nuclear magnetic resonance: A spectroscopic probe to understand the electronic structure and reactivity of molecules and materials. *J. Phys. Chem. Lett.* **12**, 2072–2085 (2021).

7. L. R. Becerra, G. J. Gerfen, R. J. Temkin, D. J. Singel, R. G. Griffin, Dynamic nuclear polarization with a cyclotron resonance maser at 5 T. *Phys. Rev. Lett.* **71**, 3561–3564 (1993).

8. D. A. Hall, D. C. Maus, G. J. Gerfen, S. J. Inati, L. R. Becerra, F. W. Dahlquist, R. G. Griffin, Polarization-enhanced NMR spectroscopy of biomolecules in frozen solution. *Science* **276**, 930–932 (1997).

9. A. B. Barnes, G. De Paëpe, P. C. A. van der Wel, K.-N. Hu, C.-G. Joo, V. S. Bajaj, M. L. Mak-Jurkauskas, J. R. Sirigiri, J. Herzfeld, R. J. Temkin, R. G. Griffin, High-field dynamic nuclear polarization for solid and solution biological NMR. *Appl. Magn. Reson.* **34**, 237–263 (2008).

10. A. J. Rossini, A. Zagdoun, M. Lelli, A. Lesage, C. Copéret, L. Emsley, Dynamic nuclear polarization surface enhanced NMR spectroscopy. *Acc. Chem. Res.* **46**, 1942–1951 (2013).

11. D. Lee, S. Hediger, G. De Paëpe, Is solid-state NMR enhanced by dynamic nuclear polarization? *Solid State Nucl. Magn. Reson.* **66-67**, 6–20 (2015).

12. D. Lee, E. Bouleau, P. Saint-Bonnet, S. Hediger, G. De Paëpe, Ultra-low temperature MAS-DNP. *J. Magn. Reson.* **264**, 116–124 (2016).

13. D. Lee, F. Mentink-Vigier, "Dynamic nuclear polarization for solid-state NMR spectroscopy," in *Modern NMR Crystallography: Concepts and Applications* (Royal Society of Chemistry, 2025), vol. 36, 256–308.

14. B. Corzilius, High-field dynamic nuclear polarization. *Annu. Rev. Phys. Chem.* **71**, 143–170 (2020).

15. R. W. Hooper, B. A. Klein, V. K. Michaelis, Dynamic nuclear polarization (DNP) 101: A new era for materials. *Chem. Mater.* **32**, 4425–4430 (2020).

16. S. Hediger, D. Lee, F. Mentink-Vigier, G. de Paëpe, MAS-DNP enhancements: Hyperpolarization, depolarization, absolute sensitivity. *eMagRes* **7**, 105–116 (2018).

17. Q. Z. Ni, E. Daviso, T. V. Can, E. Markhasin, S. K. Jawla, T. M. Swager, R. J. Temkin, J. Herzfeld, R. G. Griffin, High frequency dynamic nuclear polarization. *Acc. Chem. Res.* **46**, 1933–1941 (2013).

18. R. G. Griffin, T. M. Swager, R. J. Temkin, High frequency dynamic nuclear polarization: New directions for the 21st century. *J. Magn. Reson.* **306**, 128–133 (2019).

19. A. N. Smith, K. Märker, S. Hediger, G. De Paëpe, Natural isotopic abundance ^{13}C and ^{15}N multidimensional solid-state NMR enabled by dynamic nuclear polarization. *J. Phys. Chem. Lett.* **10**, 4652–4662 (2019).

20. K. Märker, M. Pingret, J.-M. Mouesca, D. Gasparutto, S. Hediger, G. De Paëpe, A new tool for NMR crystallography: Complete ^{13}C/^{15}N assignment of organic molecules at natural isotopic abundance using DNP-enhanced solid-state NMR. *J. Am. Chem. Soc.* **137**, 13796–13799 (2015).

21. H. Takahashi, D. Lee, L. Dubois, M. Bardet, S. Hediger, G. De Paëpe, Rapid natural-abundance 2D ^{13}C-^{13}C correlation spectroscopy using dynamic nuclear polarization enhanced solid-state NMR and matrix-free sample preparation. *Angew. Chem. Int. Ed. Engl.* **51**, 11766–11769 (2012).

22. A. J. Rossini, A. Zagdoun, F. Hegner, M. Schwarzwälder, D. Gajan, C. Copéret, A. Lesage, L. Emsley, Dynamic nuclear polarization NMR spectroscopy of microcrystalline solids. *J. Am. Chem. Soc.* **134**, 16899–16908 (2012).

23. H. Takahashi, B. Viverge, D. Lee, P. Rannou, G. De Paëpe, Towards structure determination of self-assembled peptides using dynamic nuclear polarization enhanced solid-state NMR spectroscopy. *Angew. Chem. Int. Ed. Engl.* **52**, 6979–6982 (2013).

24. R. Harrabi, T. Halbritter, F. Aussenac, O. Dakhlaoui, J. van Tol, K. K. Damodaran, D. Lee, S. Paul, S. Hediger, F. Mentink-Vigier, S. T. Sigurdsson, G. De Paëpe, Highly efficient polarizing agents for MAS-DNP of proton-dense molecular solids. *Angew. Chem. Int. Ed. Engl.* **61**, e202114103 (2022).

25. H. Takahashi, S. Hediger, G. De Paëpe, Matrix-free dynamic nuclear polarization enables solid-state NMR ^{13}C-^{13}C correlation spectroscopy of proteins at natural isotopic abundance. *Chem. Commun.* **49**, 9479–9481 (2013).

26. G. Mollica, M. Dekhil, F. Ziarelli, P. Thureau, S. Viel, Quantitative structural constraints for organic powders at natural isotopic abundance using dynamic nuclear polarization solid-state NMR spectroscopy. *Angew. Chem. Int. Ed. Engl.* **54**, 6028–6031 (2015).

27. A. N. Smith, K. Märker, T. Piretra, J. C. Boatz, I. Matlahov, R. Kodali, S. Hediger, P. C. A. van der Wel, G. De Paëpe, Structural fingerprinting of protein aggregates by dynamic nuclear polarization-enhanced solid-state NMR at natural isotopic abundance. *J. Am. Chem. Soc.* **140**, 14576–14580 (2018).

28. S. Wi, N. Dwivedi, R. Dubey, F. Mentink-Vigier, N. Sinha, Dynamic nuclear polarization-enhanced, double-quantum filtered ^{13}C-^{13}C dipolar correlation spectroscopy of natural ^{13}C abundant bone-tissue biomaterial. *J. Magn. Reson.* **335**, 107144 (2022).

29. N. Dwivedi, B. Patra, F. Mentink-Vigier, S. Wi, N. Sinha, Unveiling charge-pair salt-bridge interaction between GAGs and collagen protein in cartilage: Atomic evidence from DNP-enhanced ssNMR at natural isotopic abundance. *J. Am. Chem. Soc.* **146**, 23663–23668 (2024).

30. F. A. Perras, H. Luo, X. Zhang, N. S. Mosier, M. Pruski, M. M. Abu-Omar, Atomic-level structure characterization of biomass pre- and post-lignin treatment by dynamic nuclear polarization-enhanced solid-state NMR. *J. Phys. Chem. A* **121**, 3, 623–630 (2017).

31. A. Kirui, Z. Ling, X. Kang, M. C. Dickwella Widanage, F. Mentink-Vigier, A. D. French, T. Wang, Atomic resolution of cotton cellulose structure enabled by dynamic nuclear polarization solid-state NMR. *Cellul.* **26**, 329–339 (2019).

32. A. Kumar, B. Watbled, I. Baussanne, S. Hediger, M. Demeunynck, G. De Paëpe, Optimizing chemistry at the surface of prodrug-loaded cellulose nanofibrils with MAS-DNP. *Commun. Chem.* **6**, 58 (2023).

33. A. Kumar, H. Durand, E. Zeno, C. Balsollier, B. Watbled, C. Sillard, S. Fort, I. Baussanne, N. Belgacem, D. Lee, S. Hediger, M. Demeunynck, J. Bras, G. De Paëpe, The surface chemistry of a nanocellulose drug carrier unravelled by MAS-DNP. *Chem. Sci.* **11**, 3868–3877 (2020).

34. P. Berruyer, M. Gericke, P. Moutzouri, D. Jakobi, M. Bardet, L. Karlson, S. Schantz, T. Heinze, L. Emsley, Advanced characterization of regioselectively substituted methylcellulose model compounds by DNP enhanced solid-state NMR spectroscopy. *Carbohydr. Polym.* **262**, 117944 (2021).

35. F. Deligey, M. A. Frank, S. H. Cho, A. Kirui, F. Mentink-Vigier, M. T. Swulius, B. T. Nixon, T. Wang, Structure of in vitro-synthesized cellulose fibrils viewed by cryo-electron tomography and ^{13}C natural-abundance dynamic nuclear polarization solid-state NMR. *Biomacromolecules* **23**, 2290–2301 (2022).

36. T. Kobayashi, I. I. Slowing, M. Pruski, Measuring long-range ^{13}C-^{13}C correlations on a surface under natural abundance using dynamic nuclear polarization-enhanced solid-state nuclear magnetic resonance. *J. Phys. Chem. C* **121**, 24687–24691 (2017).

37. K. Märker, S. Paul, C. Fernández-de-Alba, D. Lee, J.-M. Mouesca, S. Hediger, G. De Paëpe, Welcoming natural isotopic abundance in solid-state NMR: Probing π-stacking and supramolecular structure of organic nanoassemblies using DNP. *Chem. Sci.* **8**, 974–987 (2017).

38. C. Sauvée, M. Rosay, G. Casano, F. Aussenac, R. T. Weber, O. Ouari, P. Tordo, Highly efficient, water-soluble polarizing agents for dynamic nuclear polarization at high frequency. *Angew. Chem. Int. Ed. Engl.* **52**, 10858–10861 (2013).

39. F. Mentink-Vigier, S. Paul, D. Lee, A. Feintuch, S. Hediger, S. Vega, G. De Paëpe, Nuclear depolarization and absolute sensitivity in magic-angle spinning cross effect dynamic nuclear polarization. *Phys. Chem. Chem. Phys.* **17**, 21824–21836 (2015).

40. F. Mentink-Vigier, I. Marin-Montesinos, A. P. Jagtap, T. Halbritter, J. van Tol, S. Hediger, D. Lee, S. T. Sigurdsson, G. De Paëpe, Computationally assisted design of polarizing agents for dynamic nuclear polarization enhanced NMR: The AsymPol family. *J. Am. Chem. Soc.* **140**, 11013–11019 (2018).

41. A. Lund, G. Casano, G. Menzildjian, M. Kaushik, G. Stevanato, M. Yulikov, R. Jabbour, D. Wisser, M. Renom-Carrasco, C. Thieuleux, F. Bernada, H. Karoui, D. Siri, M. Rosay, I. V. Sergeyev, D. Gajan, M. Lelli, L. Emsley, O. Ouari, A. Lesage, TinyPols: A family of water-soluble binitroxides tailored for dynamic nuclear polarization enhanced NMR spectroscopy at 18.8 and 21.1 T. *Chem. Sci.* **11**, 2810–2818 (2020).

42. R. Yao, D. Beriashvili, W. Zhang, S. Li, A. Safeer, A. Gurinov, A. Rockenbauer, Y. Yang, Y. Song, M. Baldus, Y. Liu, Highly bioresistant, hydrophilic and rigidly linked trityl-nitroxide biradicals for cellular high-field dynamic nuclear polarization. *Chem. Sci.* **13**, 14157–14164 (2022).

43. R. Wei, G. Casano, Y. Zhang, I. M. Gierbolini-Colón, Y. Rao, S. S. Gunaga, F. J. Scott, J. Zhou, S. Chatterjee, S. Kumari, H. Karoui, M. Huang, S. T. Sigurdsson, G. De Paëpe, Y. Liu, F. Mentink-Vigier, A. Venkatesh, O. Ouari, L. Emsley, Systematic evaluation of polarizing agents for dynamic nuclear polarization enhanced NMR. *Angew. Chem. Int. Ed.* **64**, e202505944 (2025).

44. S. Paul, E. Bouleau, Q. Reynard-Feytis, J.-P. Arnaud, F. Bancel, B. Rollet, P. Dalban-Moreynas, C. Reiter, A. Purea, F. Engelke, S. Hediger, G. De Paëpe, Sustainable and cost-effective MAS DNP-NMR at 30 K with cryogenic sample exchange. *J. Magn. Reson.* **356**, 107561 (2023).

45. Y. Matsuki, S. Nakamura, S. Fukui, H. Suematsu, T. Fujiwara, Closed-cycle cold helium magic-angle spinning for sensitivity-enhanced multi-dimensional solid-state NMR. *J. Magn. Reson.* **259**, 76–81 (2015).

46. F. Hobo, Y. Tanimoto, Y. Endo, Y. Matsuki, H. Takahashi, 400 MHz/263 GHz ultra-low temperature MAS-DNP using a closed-cycle helium gas cooling system and a solid-state microwave source. *J. Magn. Reson.* **373**, 107842 (2025).

47. Y. Matsuki, S. Nakamura, F. Hobo, Y. Endo, H. Takahashi, H. Suematsu, T. Fujiwara, Cryogenic signal amplification combined with helium-temperature MAS DNP toward ultimate NMR sensitivity at high field conditions. *J. Magn. Reson.* **335**, 107139 (2022).

48. Y. Matsuki, T. Fujiwara, "Cryogenic platforms and optimized DNP sensitivity," in *eMagRes* (John Wiley & Sons Ltd, 2018), pp. 9–24.

49. K. Thurber, R. Tycko, Low-temperature dynamic nuclear polarization with helium-cooled samples and nitrogen-driven magic-angle spinning. *J. Magn. Reson.* **264**, 99–106 (2016).

50. A. F. Mehlkopf, D. Korbee, T. A. Tiggelman, R. Freeman, Sources of t_1 noise in two-dimensional NMR. *J. Magn. Reson.* **58**, 315–323 (1984).

51. C. P. Jaroniec, C. Filip, R. G. Griffin, 3D TEDOR NMR Experiments for the simultaneous measurement of multiple carbon-nitrogen distances in uniformly ^{13}C,^{15}N-labeled solids. *J. Am. Chem. Soc.* **124**, 10728–10742 (2002).

52. G. A. Morris, H. Barjat, T. J. Horne, Reference deconvolution methods. *Prog. Nucl. Magn. Reson. Spectrosc.* **31**, 197–257 (1997).

53. A. Wokaun, R. R. Ernst, Selective detection of multiple quantum transitions in NMR by two-dimensional spectroscopy. *Chem. Phys. Lett.* **52**, 407–412 (1977).

54. A. Bax, R. Freeman, T. A. Frenkiel, An NMR technique for tracing out the carbon skeleton of an organic molecule. *J. Am. Chem. Soc.* **103**, 2102–2104 (1981).

55. T. Halbritter, R. Harrabi, S. Paul, J. van Tol, D. Lee, S. Hediger, S. T. Sigurdsson, F. Mentink-Vigier, G. De Paëpe, PyrroTriPol: A semi-rigid trityl-nitroxide for high field dynamic nuclear polarization. *Chem. Sci.* **14**, 3852–3864 (2023).

56. G. Bodenhausen, P. H. Bolton, Elimination of flip-angle effects in two-dimensional NMR spectroscopy. Application to cyclic nucleotides. *J. Magn. Reson.* **39**, 399–412 (1980).

57. R. Baumann, G. Wider, R. R. Ernst, K. Wüjthrich, Improvement of 2D NOE and 2D correlated spectra by symmetrization. *J. Magn. Reson.* **44**, 402–406 (1981).

58. G. A. Morris, Compensation of instrumental imperfections by deconvolution using an internal reference signal. *J. Magn. Reson.* **80**, 547–552 (1988).

59. A. Gibbs, G. A. Morris, Reference deconvolution: Elimination of distortions arising from reference line truncation. *J. Magn. Reson.* **91**, 77–83 (1991).

60. A. Gibbs, G. A. Morris, A. G. Swanson, D. Cowburn, Suppression of t_1 noise in 2D NMR spectroscopy by reference deconvolution. *J. Magn. Reson.* **101**, 351–356 (1993).

61. C. Brissac, T. E. Malliavin, M. A. Delsuc, Use of the cadzow procedure in 2D NMR for the reduction of t_1 noise. *J. Biomol. NMR* **6**, 361–365 (1995).

62. S. Poulding, A. J. Charlton, J. Donarski, J. C. Wilson, Removal of t_1 noise from metabolomic 2D ^1H-^{13}C HSQC NMR spectra by correlated trace denoising. *J. Magn. Reson.* **189**, 190–199 (2007).

63. L. Song, J. Wang, X. Su, X. Zhang, C. Li, X. Zhou, D. Yang, B. Jiang, M. Liu, REAL-t_1, an effective approach for t_1-noise suppression in NMR spectroscopy based on resampling algorithm. *Chin. J. Chem.* **38**, 77–81 (2020).

64. D. Koprivica, R. P. Martinho, M. Novakovic, M. J. Jaroszewicz, L. Frydman, A denoising method for multidimensional magnetic resonance spectroscopy and imaging based on compressed sensing. *J. Magn. Reson.* **338**, 107187 (2022).

65. S. Wei, Y. Ding, K. Song, Z. Liu, A robust t_1 noise suppression method in NMR spectroscopy. *Magn. Reson. Chem.* **61**, 473–480 (2023).

66. P. J. Bowyer, A. G. Swanson, G. A. Morris, Randomized acquisition for the suppression of systematic F_1 artifacts in two-dimensional NMR spectroscopy. *J. Magn. Reson.* **140**, 513–515 (1999).

67. H. Mo, J. S. Harwood, D. Yang, C. B. Post, A simple method for NMR t_1 noise suppression. *J. Magn. Reson.* **276**, 43–50 (2017).

68. Y. Nishiyama, V. Agarwal, R. Zhang, t_1-noise suppression by γ-free recoupling sequences in solid-state NMR for structural characterization of fully protonated molecules at fast MAS. *J. Phys. Chem. C* **124**, 26332–26343 (2020).

69. A. Venkatesh, X. Luan, F. A. Perras, I. Hung, W. Huang, A. J. Rossini, t_1-noise eliminated dipolar heteronuclear multiple-quantum coherence solid-state NMR spectroscopy. *Phys. Chem. Chem. Phys.* **22**, 20815–20828 (2020).

70. F. A. Perras, T. W. Goh, W. Huang, t_1-noise elimination by continuous chemical shift anisotropy refocusing. *Solid State Nucl. Magn. Reson.* **120**, 101807 (2022).

71. A. J. Robertson, M. K. Pandey, A. Marsh, Y. Nishiyama, S. P. Brown, The use of a selective saturation pulse to suppress t_1 noise in two-dimensional ^{1}H fast magic angle spinning solid-state NMR spectroscopy. *J. Magn. Reson.* **260**, 89–97 (2015).

72. Y. Ishii, J. P. Yesinowski, R. Tycko, Sensitivity enhancement in solid-state ^{13}C NMR of synthetic polymers and biopolymers by ^{1}H NMR detection with high-speed magic angle spinning. *J. Am. Chem. Soc.* **123**, 2921–2922 (2001).

73. M. Shen, S. Wegner, J. Trébosc, B. Hu, O. Lafon, J. P. Amoureux, Minimizing the t_1-noise when using an indirect ^{1}H high-resolution detection of unlabeled samples. *Solid State Nucl. Magn. Reson.* **87**, 111–116 (2017).

74. F. A. Perras, M. Pruski, Reducing t_1 noise through rapid scanning. *J. Magn. Reson.* **298**, 31–34 (2019).

75. T. J. Horne, G. A. Morris, P-Type gradient-enhanced COSY experiments show lower t_1 noise than N-type. *Magn. Reson. Chem.* **35**, 680–686 (1997).

76. W. F. Reynolds, R. G. Enriquez, Gradient-selected versus phase-cycled HMBC and HSQC: Pros and cons. *Magn. Reson. Chem.* **39**, 531–538 (2001).

77. W. E. Maas, F. H. Laukien, D. G. Cory, Gradient, high resolution, magic angle sample spinning NMR. *J. Am. Chem. Soc.* **118**, 13085–13086 (1996).

78. T. M. Alam, J. E. Jenkins, "Spectroscopy is the study of absorption and emission of electromagnetic radiation due to the interaction between matter and energy," in *Advanced Aspects of Spectroscopy* (Intech, 2012), pp. 279–306.

79. C. A. Fyfe, J. Skibsted, H. Grondey, H. Meyer zu Altenschildesche, Pulsed field gradient multiple-quantum MAS NMR spectroscopy of half-integer spin quadrupolar nuclei. *Chem. Phys. Lett.* **281**, 44–48 (1997).

80. O. W. Sørensen, M. Rance, R. R. Ernst, *z* filters for purging phase- or multiplet-distorted spectra. *J. Magn. Reson.* **56**, 527–534 (1984).

81. J. G. Pelton, D. E. Wemmer, Heteronuclear NMR pulse sequences applied to biomolecules. *Annu. Rev. Phys. Chem.* **46**, 139–168 (1995).

82. U. R. Prabhu, S. R. Chaudhari, N. Suryaprakash, Visualization of enantiomers and determination of homo- and hetero-nuclear residual dipolar and scalar couplings: The natural abundant ^{13}C edited J/D-resolved NMR techniques. *Chem. Phys. Lett.* **500**, 334–341 (2010).

83.. Kupče, T. D. W. Claridge, Molecular structure from a single NMR supersequence. *Chem. Commun.* **54**, 7139–7142 (2018).

84.. Kupče, T. D. W. Claridge, New NOAH modules for structure elucidation at natural isotopic abundance. *J. Magn. Reson.* **307**, 106568 (2019).

85. A. Lesage, C. Auger, S. Caldarelli, L. Emsley, Determination of through-bond carbon-carbon connectivities in solid-state NMR using the INADEQUATE experiment. *J. Am. Chem. Soc.* **119**, 7867–7868 (1997).

86. A. Lesage, M. Bardet, L. Emsley, Through-bond carbon-carbon connectivities in disordered solids by NMR. *J. Am. Chem. Soc.* **121**, 10987–10993 (1999).

87. S. Cadars, J. Sein, L. Duma, A. Lesage, T. N. Pham, J. H. Baltisberger, S. P. Brown, L. Emsley, The refocused INADEQUATE MAS NMR experiment in multiple spin-systems: Interpreting observed correlation peaks and optimising lineshapes. *J. Magn. Reson.* **188**, 24–34 (2007).

88. K. Takegoshi, S. Nakamura, T. Terao, ^{13}C–^{1}H dipolar-assisted rotational resonance in magic-angle spinning NMR. *Chem. Phys. Lett.* **344**, 631–637 (2001).

89. G. De Paëpe, Dipolar recoupling in magic angle spinning solid-state nuclear magnetic resonance. *Annu. Rev. Phys. Chem.* **63**, 661–684 (2012).

90. A. Brinkmann, M. Edén, M. H. Levitt, Synchronous helical pulse sequences in magic-angle spinning nuclear magnetic resonance: Double quantum recoupling of multiple-spin systems. *J. Chem. Phys.* **112**, 8539–8554 (2000).

91. M. Carravetta, M. Edén, X. Zhao, A. Brinkmann, M. H. Levitt, Symmetry principles for the design of radiofrequency pulse sequences in the nuclear magnetic resonance of rotating solids. *Chem. Phys. Lett.* **321**, 205–215 (2000).

92. A. Brinkmann, M. H. Levitt, Symmetry principles in the nuclear magnetic resonance of spinning solids: Heteronuclear recoupling by generalized Hartmann-Hahn sequences. *J. Chem. Phys.* **115**, 357–384 (2001).

93. G. Pileio, M. Concistrè, N. McLean, A. Gansmüller, R. C. D. Brown, M. H. Levitt, Analytical theory of γ-encoded double-quantum recoupling sequences in solid-state nuclear magnetic resonance. *J. Magn. Reson.* **186**, 65–74 (2007).

94. K. Saalwächter, F. Lange, K. Matyjaszewski, C.-F. Huang, R. Graf, BaBa-xy16: Robust and broadband homonuclear DQ recoupling for applications in rigid and soft solids up to the highest MAS frequencies. *J. Magn. Reson.* **212**, 204–215 (2011).

95. P. E. Kristiansen, M. Carravetta, J. D. van Beek, W. C. Lai, M. H. Levitt, Theory and applications of supercycled symmetry-based recoupling sequences in solid-state nuclear magnetic resonance. *J. Chem. Phys.* **124**, 234510 (2006).

96. Z. Zhang, H. Liu, J. Deng, R. Tycko, J. Yang, Optimization of band-selective homonuclear dipolar recoupling in solid-state NMR by a numerical phase search. *J. Chem. Phys.* **150**, 154201 (2019).

97. P. E. Kristiansen, M. Carravetta, W. C. Lai, M. H. Levitt, A robust pulse sequence for the determination of small homonuclear dipolar couplings in magic-angle spinning NMR. *Chem. Phys. Lett.* **390**, 1–7 (2004).

98. K. Märker, S. Hediger, G. De Paëpe, Efficient 2D double-quantum solid-state NMR spectroscopy with large spectral widths. *Chem. Commun.* **53**, 9155–9158 (2017).

99. M. Bak, J. T. Rasmussen, N. C. Nielsen, SIMPSON: A general simulation program for solid-state NMR spectroscopy. *J. Magn. Reson.* **213**, 366–400 (2011).

100. D. W. Juhl, Z. Tošner, T. Vosegaard, "Versatile NMR simulations using SIMPSON," in *Annual Reports on NMR Spectroscopy* (Elsevier, 2020), vol. 100, pp. 1–59.

101. M. O. Boles, R. J. Girven, The structures of ampicillin: A comparison of the anhydrate and trihydrate forms. *Acta Crystallogr. B Struct. Crystallogr. Cryst. Chem.* **32**, 2279–2284 (1976).

102. E. Bouleau, P. Saint-Bonnet, F. Mentink-Vigier, H. Takahashi, J.-F. Jacquot, M. Bardet, F. Aussenac, A. Purea, F. Engelke, S. Hediger, D. Lee, G. De Paëpe, Pushing NMR sensitivity

limits using dynamic nuclear polarization with closed-loop cryogenic helium sample spinning. *Chem. Sci.* **6**, 6806–6812 (2015).

103. D. Marion, M. Ikura, R. Tschudin, A. Bax, Rapid recording of 2D NMR spectra without phase cycling. Application to the study of hydrogen exchange in proteins. *J. Magn. Reson.* **85**, 393–399 (1989).

104. R. S. Thakur, N. D. Kurur, P. K. Madhu, Swept-frequency two-pulse phase modulation for heteronuclear dipolar decoupling in solid-state NMR. *Chem. Phys. Lett.* **426**, 459–463 (2006).

105. R. S. Thakur, N. D. Kurur, P. K. Madhu, Improved heteronuclear dipolar decoupling sequences for liquid-crystal NMR. *J. Magn. Reson.* **185**, 264–269 (2007).

106. M. Leskes, R. S. Thakur, P. K. Madhu, N. D. Kurur, S. Vega, Bimodal Floquet description of heteronuclear dipolar decoupling in solid-state nuclear magnetic resonance. *J. Chem. Phys.* **127**, 024501 (2007).

107. W. I. Goldburg, M. Lee, Nuclear magnetic resonance line narrowing by a rotating rf field. *Phys. Rev. Lett.* **11**, 255–258 (1963).
